# Towards the Development of Sustainable Hybrid Materials to Achieve High Cr(VI) Removals in a One-Pot Process

**DOI:** 10.3390/nano12223952

**Published:** 2022-11-09

**Authors:** David Gómez-Carnota, José L. Barriada, Roberto Herrero

**Affiliations:** Departamento de Química and CICA—Centro Interdisciplinar de Química e Bioloxía, Universidade da Coruña, As Carballeiras, s/n, 15071 A Coruña, Spain

**Keywords:** hybrid materials, nanostructures, chromium, one-pot reaction, reduction, adsorption, iron

## Abstract

Rising manufacturing costs resulting from the current global situation make it necessary to economize at all stages of production, including waste management. Cost-effective materials that reduce the release of pollutants into the environment are becoming mandatory. In this work, a sodium silicate polymeric material, functionalized with iron, was synthesized. The material contains iron-rich nanostructures on the surface, which are responsible for the decontamination process. The inorganic material was further treated with a reducing eucalyptus extract to improve its decontamination performance. Both the inorganic and hybrid materials were used for decontamination of Cr(VI), a widely emitted chemical waste product. The hybrid material provided the best results (1.7 g Cr(VI)·g^−1^ Fe) in a one-pot process combining reduction and adsorption. The Langmuir–Freundlich model and a statistical thermodynamics adsorption model, together with removal rates, were used to study the processes. High adsorption energies were found, especially in the adsorption of Fe(II) on the polymeric base (33.2 kJ∙mol^−1^). All materials were characterized using SEM, EDS and N_2_ sorption, TGA, and IR analyses. In conclusion, the hybrid material synthesized in this study is cheap and easy to produce through environmentally friendly synthesis, and it is a promising adsorbent for the prevention of pollution issues in effluent discharges.

## 1. Introduction

In recent years, due to the exceptional pandemic situation the world is undergoing, there has been an unprecedented increase in the cost of industrial production processes. In addition, environmental policies are becoming increasingly restrictive in order to avoid major problems in the long term. Waste treatment is unavoidable, and thus diverse strategies are being explored to increase the efficiency of waste treatment, minimizing costs and using more effective methods. Removal methods such as chemical precipitation, electrochemical reduction, ion-exchange resins, and liquid membranes have been studied and used [1,2,3,4], but they are often expensive or generate by-products. The development of new materials that are cheap to produce, either because they are synthesized from cheap and abundant raw materials [5] or because they come from waste material from other activities [6], may constitute a solution to these rising costs. Removal efficiency can be improved by synthesizing materials through a combination of several of the removal processes described above [7].

Chromium is one of the most common metals in the environment [8] due to its wide use in industrial processes, such as in the textile industry (e.g., textile dyeing, pigment production), the metalworking industry (e.g., electroplating, metal refining, refractories, etc.), in leather tanning, and in other industrial activities, such as fungicide production [9,10,11,12]. Chromium is considered the metal with the fifth most significant potential threat to health, as it is classified as a hazardous, toxic air pollutant by the ASTSDR and EPA [13,14]. Cr(VI) and Cr(III) are the main oxidation states in the Eh–pH range of natural waters. Cr(III) and its hydroxides are the main species in low Eh environments. Cr(VI) exists in solution as (Cr_2_O_7_^2−^), (CrO_4_^2−^), or (HCrO_4_^−^), depending on the pH [12,15].

Cr(VI) is considered a “human carcinogen” by the World Health Organization. It is associated with pathological effects, such as mutagenicity and genotoxicity, among others. Cr(VI) is also toxic to animals, plants, and bacteria. Cr(III) is less toxic, being an essential nutrient in small quantities, but in excess it may cause allergic reactions and even cancer [12,16].

In this work, an inorganic polymeric material was synthesized from silica-rich reagents (sodium silicate and silica colloid). This base material was doped with iron. Following this synthesis approach provides several advantages:

- A functional nanostructured surface can be obtained on a material that is simultaneously easy to handle;
- Nanoscale iron, contained in the nanostructures present on the surface of the material, is used as a decontaminating agent. This iron maintains the decontamination power associated with isolated iron nanoparticles;
- Typical problems with iron nanoparticles in solution, such as agglomeration [17], are avoided when following this approach.

The oxidation state of iron was then modified to Fe(0) using a natural extract. As a result, a hybrid material with high reducing power was obtained, without losing its surface nanostructure. This hybrid material can be used remove Cr(VI) through a process of reduction of Cr(VI) to Cr(III) and adsorption of both species in one single-pot reaction. The material is inexpensive to produce, as it is synthesized from raw materials that are abundant worldwide. The wastage of reagents is also minimal in the synthesis.

## 2. Materials and Methods

### 2.1. Chemicals

The reagents used in the studies were pure sodium silicate neutral solution and H_2_SO_4_, 95–97% expertQ, ISO, from Scharlau (Scharlab S.L., Barcelona, Spain); Ludox TM-50 colloidal silica 50 wt.% suspension in H_2_O, 1,5-diphenylcarbazide ACS, and chromium (III) chloride hexahydrate, minimum 99%, from Sigma-Aldrich (Sigma-Aldrich, Steinheim, Germany); And iron sulphate 7-hydrate PA-ACS, potassium dichromate PA-ACS-ISO, 1,10-phenantroline 1-hydrate PA-ACS, acetic acid glacial purissimum, sodium acetate 3-hydrate (RFE, USP, BP Ph. Eur.) PRS-CODEX, HCl 37% PA-ACS-ISO, NaOH 98% ACS-ISO, and hydroxylammonium chloride (ACS, ISO) from Panreac (Panreac Química S.A., Barcelona, Spain). All solutions were produced with deionized water.

### 2.2. Materials

The biomaterial used in the experiments (eucalyptus leaves) was collected in Galicia (Spain). Eucalyptus extract was prepared using an adaptation of the method described by Martinez-Cabanas et al. [18]. The eucalyptus leaves were washed with deionized water and oven-dried at 60 °C for 24 h. Then, the leaves were ground using an analytical mill, sieved, and stored. The fraction with a diameter between 0.5 and 1 mm was used to prepare the extract. 

Five grams of ground leaves was submerged in 300 mL of deionized water. Extraction was conducted by heating under reflux for 40 min. After cooling, the extract was filtered under a vacuum with a coarse filter paper. The process was first repeated using a mixed cellulose ester (MCE) filter with 1 μm pore size and subsequently with an MCE filter with 0.45 μm pore size (FILTER-LAB^®^ MCE Membrane Filter, Filtros Anoia S.A., Barcelona, Spain).

The extract had the following characteristics: reduction potential of 222 ± 6 mV vs. Ag/AgCl 3M reference electrode, pH 4.2 ± 0.2, conductivity of 430 ± 3 µS·cm^−1^, 2082 ± 73 mg·L^−1^ organic carbon, and turbidity of 54 NTU.

### 2.3. Synthesis and Optimization of the Materials

The synthesis of the base material was conducted by mixing a solution of sodium silicate in deionized water (16% *v*/*v*) with a silica colloid (Ludox TM-50) (50% sodium silicate solution and 50% Ludox) and adding different volumes of HCl to trigger the polymerization (from 6% of the solution volume to 1.5%). The mixture was poured into molds with different hole sizes and dried for 24 h. Pellets with different sizes in both diameter and thickness (from 6 × 5 mm to 2 × 1 mm) were obtained. Two types of pellet size were selected for the experiments (3 × 1 mm and 2 × 1 mm). These materials were named with the code GSLP (standing for ”granulated silica and Ludox pellets”) D3 (3 × 1 mm) and D2 (2 × 1 mm).

Several batches of pellets of the two different sizes were synthesized to optimize the dose of HCl used for polymerization. Six milliliters of the 50/50 mixture of sodium silicate and Ludox TM-50 were mixed with different amounts of HCl (from 45 to 135 μL). A complete Cr(VI) removal process was conducted with each batch as follows to choose the optimal conditions.

Both GSLP D3 and D2 were treated with an iron solution to functionalize the silicate supporting material. About 0.1 g of GSLP was submerged into 50 mL of a 250 mg·L^−1^ Fe(II) solution in conical flasks. Several flasks were agitated on an Edmund Bühler KS-15 (Edmund Bühler, Bodelshausen, Germany) rotatory shaker at 200 RPM and room temperature for 24 h. After this step, the pellets were separated from the solution by decantation. The pellets were dried on a stove (Memmert beschickung-loading model 100–800, Memmert GmbH, Schwabach, Germany) for 24 h. The codes GSLP-Fe D3 and D2 were assigned to these materials.

Both the GSLP-Fe sizes were further treated with the eucalyptus extract described in the previous section for 20 h at 25 °C to reduce the iron before using the materials in the decontamination process. The resulting materials were rinsed twice with deionized water after the treatment. These new materials were named with the codes GSLP-Fe(0) D3 and D2.

### 2.4. Measurements

Fe(II) concentration in solution was determined following a UV-VIS spectrophotometry colorimetric standard method at 510 nm [19]. A Zuzi spectrophotometer, model 4211/20 (AUXILAB, S.L., Beriáin, Spain), was used for the measurements.

Chromium (VI) measurements were also performed following a UV-VIS spectrophotometry colorimetric standard method at 540 nm [20]. Total chromium measurements were performed with flame absorption (FAAS) (Varian SpectrAA-55B) at 428.9 nm.

### 2.5. Materials Characterization

Scanning electron microscopy (SEM) (JSM-7200F microscope, Jeol Ltd., Tokio, Japan) and energy-dispersive X-ray spectroscopy (EDS) (Oxford EDS X-Max^N^ detector, Oxford Instruments, Abingdon, England) tests were conducted to examine the surfaces and the distribution of the adsorbed metals of the different materials used in the experiments. N_2_ sorption tests (Tristar II plus 3030 Surface Area and Porosity Analyzer, Micrometrics Instruments Corporation, Norcross, GA, USA) were performed to determine the porosity and adsorption surface area.

### 2.6. Kinetic Studies

Iron sorption and chromium removal reaction rates were studied for both pellet sizes. For the iron experiments, 0.2 g of GSLP was submerged into 100 mL of a 75 mg·L^−1^ Fe(II) solution. Experiments were performed at room temperature and natural pH and under agitation over 24 h. For the chromium removal studies, the influence of pH and initial chromium concentration was studied, and 0.2 g of GSLP-Fe(0) was submerged into different 100 mL chromium solutions (from 50 to 100 mg·L^−1^) acidified with HCl 4M (pH = 1.2 and 0.8). Experiments were performed at room temperature with stirring over 24 h.

### 2.7. Equilibrium Studies

Iron sorption experiments were conducted by submerging 0.1 g of both GSLP sizes in several 50 mL Fe(II) solutions with concentrations between 10 and 75 mg·L^−1^. Flasks were stirred for 24 h at room temperature and natural pH (ca. pH 5).

Chromium sorption was studied using GSLP and GSLP-Fe. The complete Cr(VI) removal process was studied using GSLP-Fe(0). For the GSLP sorption studies, 0.1 g of GSLP was added to several 50 mL Cr(VI) solutions with concentrations between 10 and 150 mg·L^−1^. For the GSLP-Fe sorption studies, 0.1 g of GSLP-Fe was submerged in 50 mL Cr(VI) solutions with concentrations between 10 and 300 mg·L^−1^. Chromium removal experiments using GSLP-Fe(0) were conducted by adding 0.1 g to 50 mL Cr(VI) solutions with concentrations between 10 and 500 mg·L^−1^. Solutions were acidified with 1 mL of a 4 M HCl solution (pH ≈ 0.8) and agitated for 24 h at room temperature.

## 3. Results and Discussion

### 3.1. Materials and Characterization

In this work, three different materials were synthesized, starting with a polymerized silicate base that was subsequently transformed with different processes.

The first material, called GSLP, was white and semi-translucent. Two types of pellets were obtained using the conditions described in Section 2.3. The largest pellets were 3 mm in diameter and 1 mm in thickness, with a powdery external aspect. This size was chosen for comparison with a similar material studied in a previous work, as pellets of that material had the same size. The code D3 was assigned to this pellet size. The smaller pellets were 2 mm in diameter and 1 mm in thickness and had a less powdery aspect. The code D2 was assigned to this pellet size. This size was chosen because it was smaller and, consequently, had a larger surface/volume ratio, and it could also be easily synthesized and handled. The purpose of this smaller size was to evaluate if Cr(VI) removal was increased with the reduction in the pellet size.

SEM and EDS analyses were performed for both GSLP sizes. The D3 pellets had a roughened surface and NaCl deposits could be seen on the silicate surface. NaCl is a by-product of the reaction between Na_2_SiO_3_ and HCl. The D2 pellets had a much smoother surface than the D3 pellets. NaCl was not found in deposits on the silicate surface but was spread evenly over the surface.

**Figure 1:**
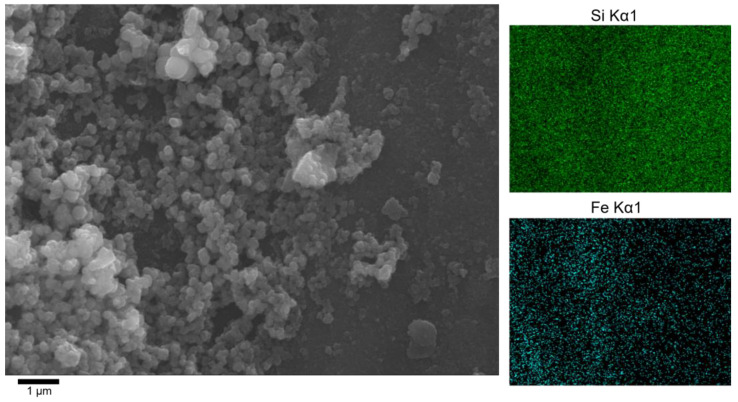
SEM image of GSLP-Fe (D3) (10,000× magnification) and EDS maps. Colored zones indicate the presence of each element: Si (green), Fe (cyan).

Chromium removal was not observed when GSLP was used in the sorption studies. This is discussed in more detail in Section 3.3.3. Due to this fact, the GSLP was transformed by the addition of Fe(II). The new material, named GSLP-Fe, had a reddish-brown surface, and the D3 pellets no longer had a powdery aspect. SEM and EDS analyses showed that there was no NaCl on the pellet surfaces, so it was concluded that it was dissolved in the Fe(II) solution used in the synthesis. No major differences between the two pellet sizes were observed. Figure 1 shows the SEM image of the GSLP-Fe and the EDS maps corresponding to the image as an example. In the SEM image, it can be seen that the material surface is nanostructured. The EDS maps indicate a greater presence of Fe in the areas where these formations are located. These nanostructures were not observed in the SEM images of the GSLP, which indicated that they were formed during the treatment of GSLP with the Fe(II) solution. The formations were responsible for the high BET surface of the material. These nanostructures were stable after the functionalization of the material and its subsequent use in the decontamination process since, as discussed later in this section, the area of the surface remained almost constant throughout the mentioned processes.

**Figure 2:**
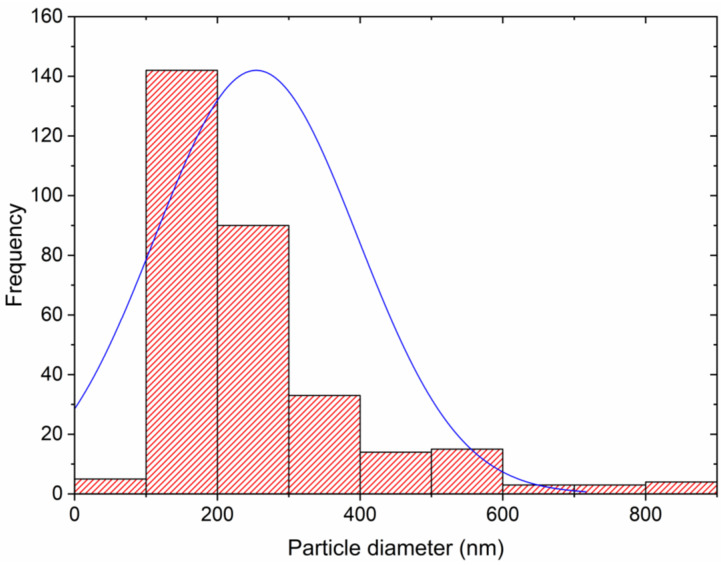
Histogram of the particle size of the GSLP-Fe surface. ▬, Normal distribution curve.

The surface of the GSLP-Fe was studied to determine the size of these structures present on it. The SEM images were analyzed using an image processing and analysis program called ImageJ [21]. Figure 2 shows the histogram obtained from these analyses. Most of the particle diameters were between 100 and 200 nm. The mean particle diameter was 254 nm, with a standard deviation of 142 nm. The normal distribution curve indicated that 99% of the particle diameters were between 0 and 680 nm. 

GSLP-Fe can remove Cr(VI) through adsorption, as discussed in Section 3.3.3. However, a second transformation of the material was performed using a eucalyptus extract as a reductant agent to increase the material removal capacity. The resulting material, GSLP-Fe(0), had a black surface. The other characteristics of the pellets remained unchanged from those of GSLP-Fe. SEM and EDS analyses also showed no significant changes compared to the GSLP-Fe analyses. There were also no significant differences between the two pellet sizes. This indicated that the eucalyptus extract only changed the oxidation state of iron, black being the characteristic color of Fe(0). In Section 2.2. it was stated that eucalyptus extract has reducing properties. During the Cr(VI) removal experiments, evidence of the change in the oxidation state of iron was also found. GSLP-Fe(0) could reduce Cr(VI) to Cr(III). This reduction was not observed when GSLP-Fe was used (see Section 3.3.3 and Section 3.3.4).

**Figure 3:**
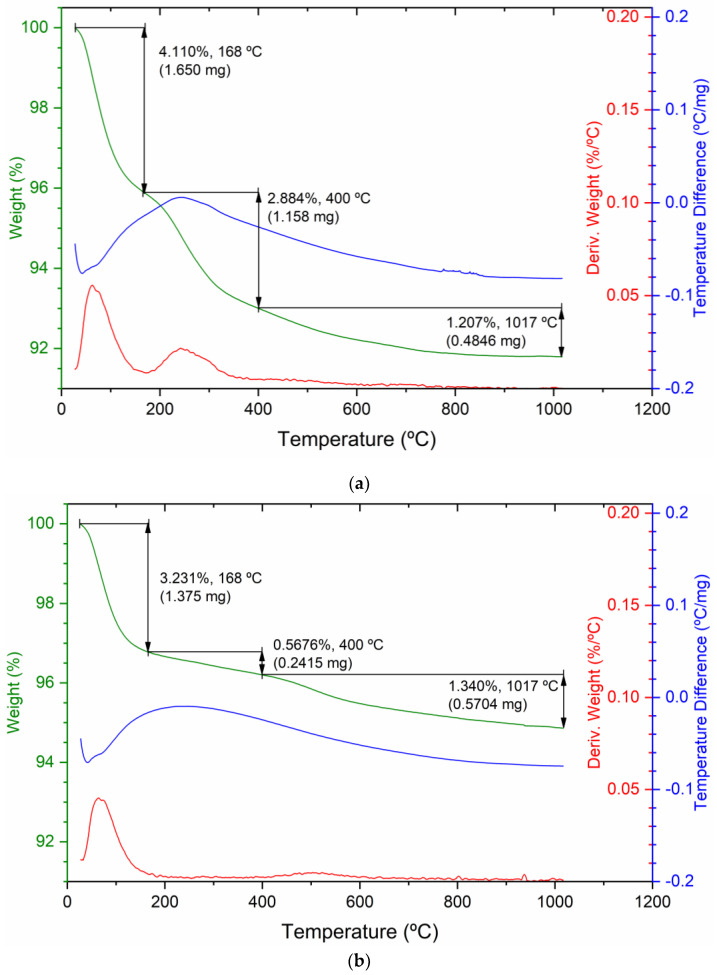
(**a**) TGA graphs of GSLP-Fe(0) D3 and (**b**) GSLP-Fe D3 decomposition in air (% weight, green). The derivative thermogravimetry (DTG, %/°C, red) and temperature difference (°C/mg, blue) are also shown for both graphs.

Thermogravimetric analyses (TGAs) were performed to check whether organic matter was adsorbed on the material during the treatment with the eucalyptus extract. GSLP-Fe(0) pellets (D3 and D2) and GSLP-Fe pellets (D3 and D2) were analyzed in the presence of air. The TGAs performed with both pellet sizes were very similar. Figure 3 shows the TGAs of GSLP-Fe(0) and GSLP-Fe D3 as examples. All TGA graphs presented a slope indicative of multi-step decomposition. All TGAs indicated a very similar mass loss (3.5–4%) from 25 to 168 °C. This mass was associated with water loss. Between 170 °C and 400 °C, a clear difference could be seen in the slopes of the TGAs. In the GSLP-Fe(0) TGA, a mass loss of 2.5–2.8% appeared, while in the GSLP-Fe TGA, no clear mass loss was observed in this temperature range. This confirmed that, during the treatment with the eucalyptus extract, a small amount of biomass was adsorbed on the material.

The gases emitted during the TGAs were analyzed to corroborate this hypothesis. The IR spectra of the four samples at a temperature of ca. 98 °C had signals from 4000 to 3500 cm^−1^ corresponding to O–H stretching. Signals corresponding to O–H bending appear at 1550–1300 cm^−1^. These signals confirmed that the mass loss between 25 and 168 °C corresponded to water loss.

In the GSLP-Fe(0) IR spectra at approximately 260 °C, intense signals appeared at 2400–2300 cm^−1^ corresponding to O=C=O stretching of CO_2_. These signals proved that, at this temperature, the organic matter adsorbed during the eucalyptus extract treatment was removed from the GSLP-Fe(0) [22,23].

**Figure 4:**
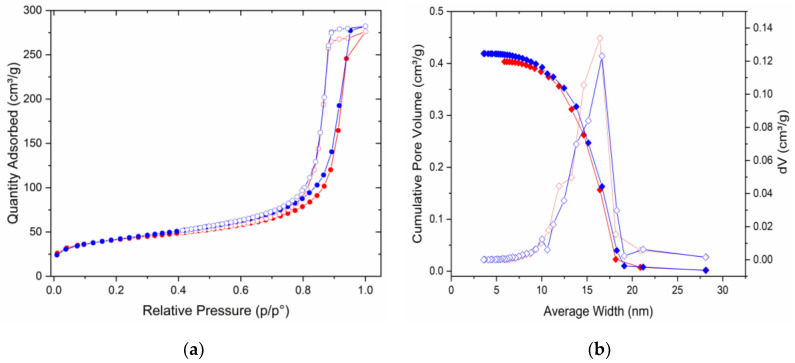
(**a**) N_2_ adsorption (filled circles) and desorption (empty circles) isotherms for GSLP-Fe(0) D2 (red) and GSLP-Fe(0) D2 chromium desorption and Fe(0) regeneration (blue). T = 77 K. (**b**) Pore size distribution (hollow diamonds) and cumulative pore volume (filled diamonds) for GSLP-Fe(0) D2 (red) and GSLP-Fe(0) D2 chromium desorption and Fe(0) regeneration (blue).

N_2_ sorption tests were performed using GSLP-Fe (D3 and D2), GSLP-Fe(0) (D3 and D2), and GSLP-Fe(0) after the Cr(VI) elimination, subsequent chromium desorption, and Fe(0) regeneration. The experimental data conformed to the type-four isotherm among those described by Brunauer et al. [24,25]. BET surfaces between 132 and 145 m^2^∙g^−1^ were found. Pore size distribution (PSD) was calculated with the Barrett–Joyner–Halenda (BJH) method. The results showed a large contribution from macropores. Figure 4 shows the BET and PSD for raw GSLP-Fe(0) D2 and the same material after chromium desorption and Fe(0) regeneration as an example. The results of all tests are summarized in Table 1. As can be seen, the smaller pellets had larger adsorption surfaces areas. Furthermore, it was remarkable that the new and regenerated GSLP-Fe(0) had almost identical surface areas, which indicates that the material could be used and regenerated without the loss of the nanostructures present on the surface of the material.

Extensive information, such as optical images of the materials (Figures S1 and S2), SEM images of the different materials discussed in this section, EDS analysis and maps (Figures S3–S13, Table S1), TGA graphs (Figure S14), gas IR spectra (Figures S15 and S16), and N_2_ sorption and PSD graphs (Figures S17–S19), can be found in the Supplementary Materials.

#### 3.1.1. GSLP Synthesis Optimization

GSLP synthesis was optimized by varying the dose of HCl used for the polymerization of the Na_2_SiO_3_–Ludox mixture for both pellet sizes. For the tests, several batches of GSLP were synthesized and subjected to the complete process of functionalization with iron, eucalyptus reduction, and Cr(VI) removal. Each test was performed in triplicate. The optimal HCl dose was chosen based on Cr(VI) removal, reproducibility, and material properties (hardness, mechanical strength, and stability in the decontamination medium). For the D3 pellets, 120 µL of 4M HCl provided very low deviation in the three trials. Higher HCl doses decreased the reproducibility, and stability problems began to appear. For the D2 pellets, a 75 µL dose of 4M HCl was used because this dose achieved the highest Cr(VI) removal with the lowest deviation.

A figure showing the results of this experiment can be found in the Supplementary Materials (Figure S20).

#### 3.1.2. Characterization of the Iron Adsorbed on GSLP-Fe

Previous studies have analyzed similar materials to the ones employed in this study using XPS. It was found that, when Fe(II) was adsorbed on the surface of an acid polymerized silicate material, rapid oxidation of Fe(II) to Fe(III) took place. After this process, the Fe present in the material was mainly Fe(III), with a small proportion of Fe(II) [26].

An adsorption experiment involving Fe(II) on GSLP was conducted in an inert atmosphere (N_2_) to corroborate this hypothesis. As long as the inert atmosphere was maintained, Fe(II) adsorption occurred as usual, and the surfaces of the pellets turned from white to blue instead of the reddish-brown color characteristic of GSLP-Fe. When the inert atmosphere was removed, the surfaces of the pellets changed from blue to reddish-brown within a few hours. The color change from white to reddish-brown that took place in the normal experiments proved that very fast Fe(II) oxidation occurred on the surface of the pellets.

Despite the oxidation of Fe(II) to Fe(III), it was preferable to use an Fe(II) solution to synthetize the GSLP-Fe. When using an Fe(III) solution, the iron sorption on the material was very limited, as the natural pH of the solution was very acidic, around pH 2.5. Using Fe(II) for the synthesis allowed the material to be prepared without modifying the pH of the medium, which meant lower production costs and less preparation time.

Using an Fe(0) suspension on GSLP to directly synthesize GSLP-Fe(0) was also not viable. Fe(0) could be deposited on GSLP. However, when the material was immersed in the acidic Cr(VI) solution, the Fe(0) was released very quickly into the solution.

#### 3.1.3. Stability of the Synthesized Materials

The stability of the materials was evaluated under the pH conditions used in the kinetic and equilibrium studies.

All materials were inert in deionized water. After one week immersed in deionized water, the pellets released less than 1% of the adsorbed iron. The materials were also mechanically resistant. Pellets withstood stirring at 200 RPM in an orbital shaker for several days without apparent degradation. 

The stability of each material was significantly different at acidic pH (pH ca. 1). The stability of GSLP was similar as that in deionized water, with no apparent degradation after several days. GSLP-Fe underwent a very slow Fe(III) desorption process at pH levels between 1 and 2, taking more than a week reach completion. The stability of GSLP-Fe(0) was much better than that of GSLP-Fe in acidic media due to the organic matter adsorbed during the treatment with the eucalyptus extract, which acted as a capping agent. GSLP-Fe(0) endured 3–4 days at pH ≥ 1 without releasing Fe(III). The degradation was accelerated at pH < 1, although the material could withstand more than 72 h at pH ≈ 0.8 without degrading.

Analyses undertaken with aqueous eucalyptus extracts showed that there was a wide variety of compounds in their composition, such as 1,8-cineole, pinene, and eucalyptol, among many others [27,28]. The Folin–Ciocalteu method revealed a considerable number of polyphenols among these compounds. By measuring the DPPH radical scavenging capacity, the compounds present in the extract were found to demonstrate important antioxidant activity [29,30]. This explained the performance of the organic matter adsorbed on GSLP-Fe(0) as a capping agent.

A fast iron release was observed for both GSLP-Fe and GSLP-Fe(0) when these materials were immersed in strong acids, such as concentrated H_2_SO_4_ or HNO_3_. The degradation was so fast that most of the iron was stripped from both materials within 12 h (H_2_SO_4_ at pH ≈ 1) or 24 h (concentrated HNO_3_).

### 3.2. Kinetic Studies

Kinetic studies were conducted to describe the kinetic processes involved in the Fe(II) adsorption and Cr(VI) removal. For the Cr(VI) removal, experiments were performed to check if the pH and adsorbate concentration influenced the reaction rate.

The rate of adsorption of Fe(II) by GSLP was fast for both pellet sizes (D3 and D2). Eighty percent of the reaction took place in the first 2.5 h, and equilibrium was reached in about 7 h. Cr(VI) elimination was slower. With the faster kinetics (D2 pellets, pH ≈ 0.8), the equilibrium was reached in about 24 h, while with the slower kinetics, the reaction continued to progress after 72 h. With the slower kinetics, 80% of the reaction was exceeded in 24 h.

#### 3.2.1. Kinetic Models

Two diffusion models were applied to describe the experimental data, the Webber and Morris model [31] and the Boyd model [32].

To determine the rate controlling step of reactions, sorption processes can be described in three stages: diffusion from the solution to the adsorbent (bulk diffusion), diffusion from the film to the adsorbent surface (film diffusion), and diffusion through the adsorbent particles (intraparticle diffusion). For a reduction process, the stages are similar, with the last stage being diffusion through the reductant particles. Bulk diffusion is minimal with efficient stirring. Therefore, this process rate is usually governed by intraparticle or film diffusion.

The Webber and Morris model is a simple model that is easy to apply using the following equation:

(1) $$q_{t}{=k}_{i}t^{½}+C$$

where q_t_ is the metal amount adsorbed (mg·g^−1^) at time t (h), and k_i_ is the intraparticle constant (mg·g^−1^·h^−½^). This model was applied to test if the diffusion model fit the experimental data.

The Boyd model was used to study the mechanism in more detail. The Boyd model describes particle diffusion with the following equation:

(2) $$F=1\;-\;\frac{6}{\pi^{2}}\overset{\infty }{\underset{n=1}{\sum }}\frac{1}{n^{2}}\exp\left(-\frac{D_{i}\pi^{2}n^{2}t}{r^{2}}\right)$$

where F is the fraction of metal removed at time t, defined by the following expression:

(3) $$F=\frac{{\;q}_{t}}{q}$$

where q_t_ is the amount of metal removed at time t and q is the amount of metal removed at the equilibrium. The Boyd model also defines a time constant B, given by:

(4) $$B=\frac{D_{i}\pi^{2}}{r^{2}}$$

where D_i_ is the effective diffusion coefficient of the metal in the solid phase (sorbent or reductant in this case) (cm^2^·h^−1^) and r is the radius of the solid particle (cm).

The Boyd model can also be applied by performing a linearization of Equation (2). Reichenberg [33] explains that, at sufficiently high values of F, only one term of the series needs to be used. When this is applied and B is substituted into Equation (2), the following expression is obtained:

(5) $$F=1\;-\frac{6}{\pi^{2}}\exp\left(-\mathrm{Bt}\right)\;;\;\mathrm{Bt}=-\;\ln\left(\frac{\pi^{2}}{6}\left(1\;-\;F\right)\right)=-\;0.4977\;-\;\ln\left(1\;-\;F\right)$$

This equation gives small error values for Bt when F ≈ 1, but it provides large error values at low F values, increasing to − 0.4977 when F = 0. To correct this, for the lower range of values of F (F < 0.86), Equation (5) is transformed into:

(6) $$\mathrm{Bt}=2\pi \;-\frac{\pi^{2}F}{3}-\;2\pi {\left(1\;-\frac{\pi F}{3}\right)}^{\text{½}}$$

which minimize the error for F at low values [33].

The linearized Boyd model provides useful information. If the fitting of Bt vs. t is a straight line that crosses through the origin, particle diffusion is considered the limiting step. If a straight line is obtained that does not pass through the origin, the process is governed by film diffusion.

#### 3.2.2. Iron Sorption Kinetics

**Figure 5:**
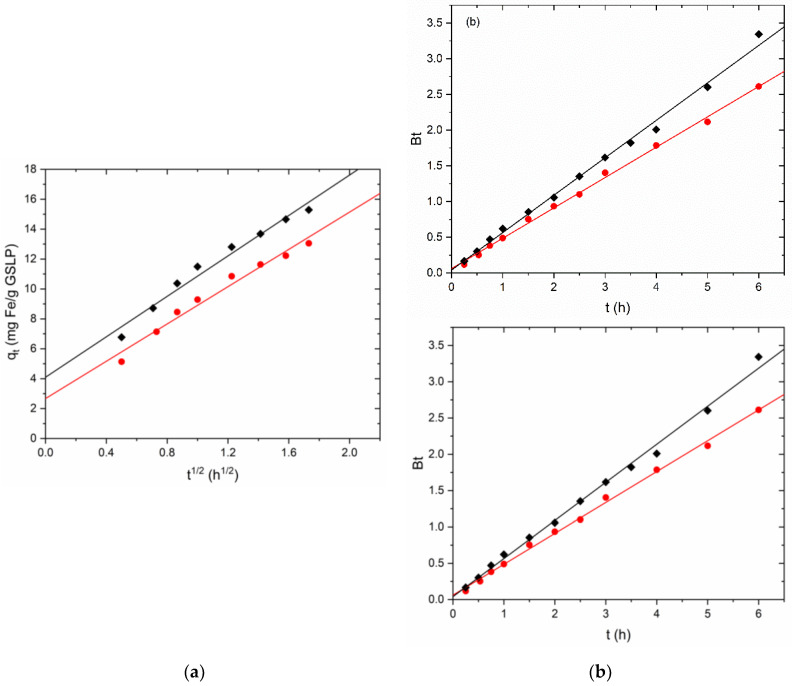
(**a**) Linearized adsorption kinetics of Fe(II) sorption by GSLP D3 (●) and GSLP D2 (♦) using the Webber–Morris model. (**b**) Linearized Fe adsorption kinetics of Fe(II) sorption by GSLP D3 (●) and GSLP D2 (♦) using the Boyd model. GSLP dose of 2 g·L^−1^, natural pH (ca. 5), room temperature, and stirring at 200 RPM.

Figure 5a shows the fitting data according to the Webber and Morris model. The parameters obtained from the fitting are summarized in Table 2 together with their associated errors. As can be seen, the model describes the data obtained in the kinetics quite well. This indicates that the adsorption of Fe(II) is a diffusion-governed process.

As mentioned in the previous section, the Boyd model was used to study the process in more detail. The nonlinear model (Equation (2) with Equations (3) and (4) substituted) was applied to the experimental data with good results. The model correctly described the experimental data, obtaining R^2^ values of 0.996–0.997. 

The linear Boyd model was used to determine the limiting step. Figure 5b shows the Bt vs. t fitting with the Bt data obtained from Equations (5) and (6). Table 2 shows the values acquired for the intercept obtained from the fitting. The value of the intercept and its error for GSLP D2 included the origin of coordinates, so particle diffusion was considered the limiting step. For GSLP D3, Student’s t-test was used to determine whether the intercept was statistically 0. In this case, at 95% confidence, the ordinate was not statistically 0, although it was at 99%, so it was concluded that the process was governed by both particle and film diffusion.

#### 3.2.3. Chromium Removal Kinetics

The Webber and Morris model was first used to study Cr(VI) removal kinetics. Using this model, two different linear trends could be observed with a nonlinear zone between them. This distribution of the experimental points was consistent with the structure in two different steps of the Cr(VI) removal process using GSLP-Fe(0). As explained in Section 3.3.4, the Cr(VI) removal process using GSLP-Fe(0) consisted of two steps: reduction of Cr(VI) to Cr(III) and adsorption of both species onto the material. The first linear trend appeared during the first three hours for all kinetics performed. In this part of the process, reduction of Cr(VI) to Cr(III) predominated. The second linear trend began after 7 h of reaction for kinetics lasting longer than 24 h. In this part, the predominant process was the chromium adsorption. Between the two linear trends, a nonlinear zone appeared in which neither of the two processes predominated over the other. In the case of the fastest kinetics, where equilibrium was reached in about 24 h, this nonlinear zone was not observed because the reaction was faster.

The parameters of the fitting of both trends can be seen in Table 2. For the linear trends corresponding to the reduction step, straight lines with r 0.979–0.999 were obtained. For the adsorption step, straight lines with good r (0.964–0.996) were also obtained. These results indicate that the Cr(VI) removal process was governed by diffusion.

**Figure 6:**
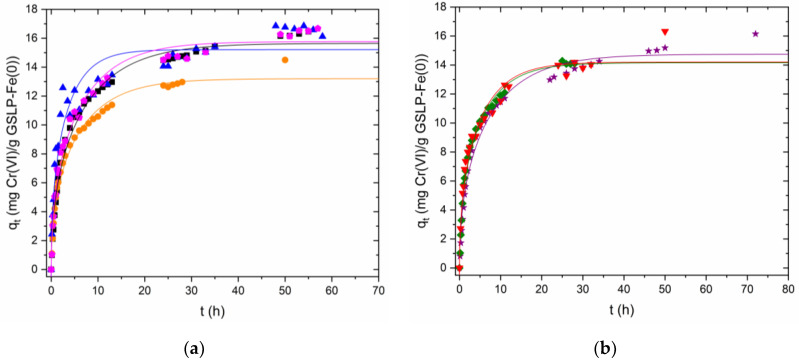
(**a**) Kinetic experimental data for Cr(VI) removal by GSLP-Fe(0) D3 fitted using the nonlinear Boyd model. The GSLP-Fe(0) D3 dose was 2 g·L^−1^, equaling 27.8 mg·L^−1^ of Fe, and room temperature and stirring at 200 RPM were used for all experiments. All experiments were acidified using HCl 4M. (■) pH ≈ 1.2, initial Cr(VI) concentration: 50 mg·L^−1^; (●) pH ≈ 0.8, initial Cr(VI) concentration: 50 mg·L^−1^; (⬟) pH ≈ 0.8, initial Cr(VI) concentration: 75 mg·L^−1^; (▲) pH ≈ 0.8, initial Cr(VI) concentration: 100 mg·L^−1^. (**b**) Kinetic experimental data for Cr(VI) removal by GSLP-Fe(0) D2 fitted using the Boyd model. The GSLP-Fe(0) D2 dose was 2 g·L^−1^, equaling 32.5 mg·L^−1^ of Fe, and room temperature and stirring at 200 RPM were used for all experiments. All experiments were acidified using HCl 4M. (★) pH ≈ 1.2, initial Cr(VI) concentration: 50 mg·L^−1^; (♦) pH ≈ 0.8, initial Cr(VI) concentration: 50 mg·L^−1^; (▼) pH ≈ 0.8, initial Cr(VI) concentration: 75 mg·L^−1^.

The nonlinear Boyd model showed worse fittings for Cr(VI) removal kinetics than those obtained for Fe(II) sorption. The model fit the beginning of the kinetics well but did not correctly describe the trend of the slower kinetics where the reaction proceeded slowly and did not reach a plateau (Figure 6a,b). R^2^ values from 0.917 to 0.986 were obtained.

**Figure 7:**
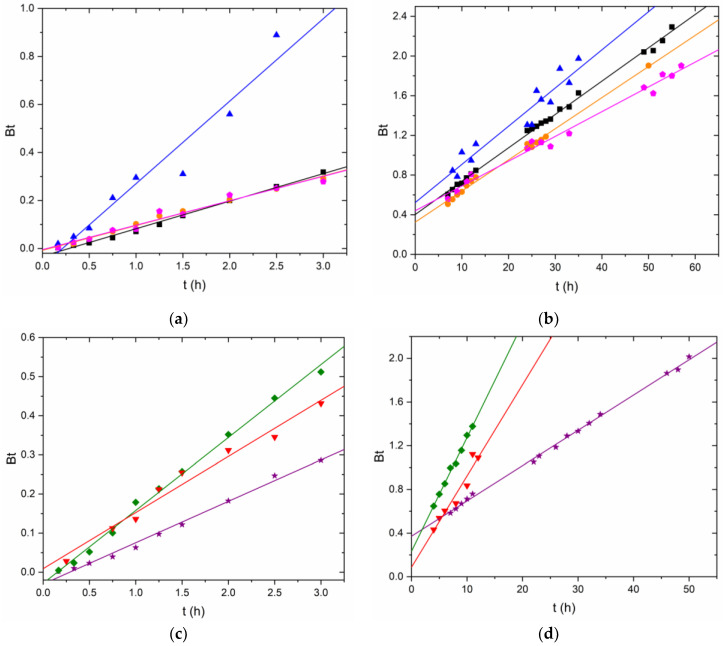
Linearized kinetics of Cr(VI) removal by GSLP-Fe(0) using the Boyd model. (**a**) First linear part of the kinetics using GSLP-Fe(0) D3. (**b**) Second linear part of the kinetics using GSLP-Fe(0) D3. (**c**) First linear part of the kinetics using GSLP-Fe(0) D2. (**d**) Second linear part of the kinetics using GSLP-Fe(0) D2. All experiments were acidified using HCl 4M. (■) pH ≈ 1.2, initial Cr(VI) concentration: 50 mg·L^−1^; (●) pH ≈ 0.8, initial Cr(VI) concentration: 50 mg·L^−1^; (⬟) pH ≈ 0.8, initial Cr(VI) concentration: 75 mg·L^−1^; (▲) pH ≈ 0.8, initial Cr(VI) concentration: 100 mg·L^−1^. (★) pH ≈ 1.2, initial Cr(VI) concentration: 50 mg·L^−1^; (♦) pH ≈ 0.8, initial Cr(VI) concentration: 50 mg·L^−1^; (▼) pH ≈ 0.8, initial Cr(VI) concentration: 75 mg·L^−1^.

The linearized Boyd model was applied to each linear trend separately (Figure 7, Table 2). Straight lines with r 0.972–0.998 in the reduction step and 0.959–0.999 in the adsorption step were obtained. Intercepts were studied using Student’s *t*-test. For the kinetics at pH 0.8, the ordinates were statistically 0 at 95% confidence, except for the kinetics of D2 at c_i_ 50 mg·L^−1^ (♦), which departed from 0 by a very small amount. For the two kinetics at pH 1.2, the ordinate was not statistically 0. From these data, it was concluded that, at pH 0.8, particle diffusion was the limiting step in the reduction stage. At pH 1.2, the lower proton concentration may have caused the reduction to be less dominant, so simultaneous processes affecting the intercept could have been present. In the adsorption step, the ordinates were not close to being statistically 0, except for the D2 c_i_ 75 mg·L^−1^ kinetics (▼). From these data, it was concluded that the adsorption step was governed by film diffusion.

After analyzing all the data, no clear influence from the initial Cr(VI) concentration on the kinetics was observed. However, pH influence was clearly observed. At pH 0.8, the reaction equilibrium was reached using GSLP-Fe(0) D2, although the Cr(VI) removal was slightly higher at pH 1.2. Since equilibrium was reached at pH 0.8 using GSLP-Fe(0) D2, to compare both pellet sizes under the same conditions, pH 0.8 and 24 h reaction time were chosen as the conditions for the elimination studies.

Extended information, such as kinetic experimental data (Figure S21), experimental data represented according to the Webber and Morris model (Figures S22 and S23), and nonlinear Boyd parameters (Table S2), can be found in the Supplementary Materials.

### 3.3. Equilibria Studies

#### 3.3.1. Isotherm Models

Two isotherm models were used for the analysis of equilibrium experiments, the Langmuir–Freundlich model and a statistical thermodynamic-based model described by Sellaoui et al. [34]. This model is very versatile, and it can be adapted to multiple types of adsorptions (monolayer, multilayer, simple, binary, etc.).

The Langmuir–Freundlich model is one of the most widely used models in adsorption studies:

(7) q=Q0bc1n1+bc1n

where q is the amount of metal adsorbed at equilibrium, c is the metal concentration in solution at equilibrium, Q_0_ is the maximum adsorption capacity, b is an affinity parameter (high b values indicate a steep beginning in the isotherm, which reflects the high affinity of the sorbent for the sorbate), and n is a parameter related to surface heterogeneity.

The Sellaoui model based on statistical thermodynamics is used under the condition of monolayer adsorption:

(8) $$q=\frac{Q_{0}}{{1+(c}_{½}⁄{c)}^{m}}$$

where q is the metal amount adsorbed at equilibrium, Q_0_ is the maximum adsorption capacity, c is the metal concentration in solution at equilibrium, c_½_ is the concentration at half saturation of the sorbent, and m is the number of atoms per adsorption site. 

Sellaoui also describes the relationship between the concentration at half saturation and the adsorption energy with the following equation [35]:

(9) $$E=\mathrm{RTln}\frac{c_{s}}{c_{½}}$$

where c_s_ is the solubility of the adsorbate.

#### 3.3.2. Iron Sorption on GSLP

The Fe(II) adsorption studies were conducted at natural pH (*ca*. pH 5). Previous studies using a similar polymeric base have shown this to be the simplest and most effective condition for this process [26]. With this condition, high adsorption is achieved, iron precipitation as hydroxide is avoided, and no time is wasted with pH modifications.

**Figure 8:**
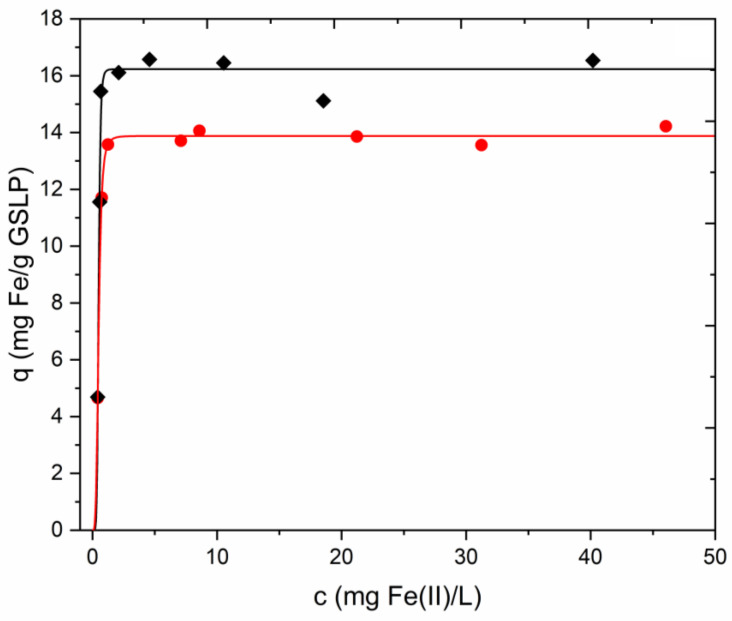
Experimental data for Fe(II) sorption on GSLP D3 (●) and GSLP D2 (♦). Solid lines were obtained by fitting with Equation (8). GSLP dose of 2 g·L^−1^, natural pH (ca. 5), room temperature, and stirring at 200 RPM. Initial Fe(II) concentration from 10 to 75 mg·L^−1^ was used.

The plots of the adsorption equilibria fitted with Equations (7) and (8) were identical. Therefore, only one fit (Equation (8)) is shown in this article. A figure showing the experimental data fitted with Equation (7) can be found in the Supplementary Materials (Figure S24). Figure 8 shows the experimental data fitted with Equation (8). The parameters obtained from the fits are summarized with their associated errors in Table 3. As can be seen, both models gave the same values for Q_0_. Moreover, b and n are the reciprocals of c_½_ and m, respectively. The adsorption energy could be calculated with Equation (9) and the parameter c_½_ obtained with Equation (8). The results obtained show the high affinity that GSLP had for Fe(II). This fact was reflected by the almost vertical slope before the plateau in the isotherm, the high values of the parameter b, and the high adsorption energy. Smaller pellets (D2) adsorbed more Fe(II) when comparing the two pellet sizes due to the larger surface area exposed.

The amount of Fe adsorbed on GSLP was confirmed by desorption. A 0.25 M HCl solution was used because it caused complete stripping of the iron present in the pellets but did not alter its oxidation state. An aliquot was also taken from the stripping solution and treated with a reduction procedure to transform any Fe(III) to Fe(II) before measuring the iron in solution. Values of 13.6 ± 0.7 mg Fe·g^−1^ GSLP for D3 pellets and 16.0 ± 0.1 mg Fe·g^−1^ GSLP for D2 pellets were found, which were consistent with the Q_0_ values obtained with Equations (7) and (8) (Table 3).

#### 3.3.3. Cr(VI) Sorption on GSLP and GSLP-Fe

Cr(VI) adsorption studies were performed using GSLP and GSLP-Fe, as the use of GSLP-Fe(0) adds a reduction step to the process.

No evidence of Cr(VI) adsorption was observed using GSLP as adsorbent. In the colorimetric measurements, values close to 0 were obtained, which varied randomly as the concentration was increased. In the SEM and EDS analyses of the GSLP used in this process, no chromium signals were found. The results show that GSLP only worked as a support for iron and was not involved in the Cr(VI) removal process. The iron present in GSLP-Fe and GSLP-Fe(0) was the species that acted as a Cr(VI) remover. Further information on chromium sorption on GSLP can be found in the Supplementary Materials (Figures S25–S27).

**Figure 9:**
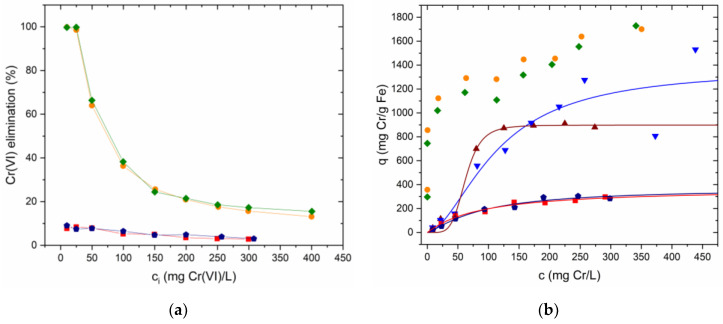
(**a**) Cr(VI) elimination percentages using GSLP-Fe(0) D3 (●) and D2 (♦). Cr(VI) elimination percentages using GSLP-Fe D3 (■) and D2 (⬟). (**b**) Equilibria experimental data. Solid lines were obtained by fitting to Equation (8). (■) Cr(VI) adsorption by GSLP-Fe D3. (⬟) Cr(VI) adsorption by GSLP-Fe D2. (●) Maximum Cr(VI) elimination by GSLP-Fe(0) D3. (♦) Maximum Cr(VI) elimination by GSLP-Fe(0) D2. (▲) Total chromium adsorption by GSLP-Fe(0) D3. (▼) Total chromium adsorption by GSLP-Fe(0) D2. The GSLP-Fe/Fe(0) D3 dose of 2 g·L^−1^ equaled 27.8 mg·L^−1^ of Fe. The GSLP-Fe/Fe(0) D2 dose of 2 g·L^−1^ equaled 32.5 mg·L^−1^ of Fe. pH 1, room temperature, and stirring at 200 RPM for all the experiments. Initial Cr(VI) concentration from 10 to 500 mg·L^−1^ was used.

Cr(VI) sorption was observed when GSLP-Fe was used as adsorbent. Figure 9a shows the Cr(VI) removal percentages achieved using GSLP-Fe with respect to the initial Cr(VI) concentration. Figure 9b shows the data in isotherm form, fitted by Equation (8) (data fitted by Equation (7) can be found in the Supplementary Materials (Figure S28)). The parameter values of the fits and their errors are listed in Table 3, along with the R^2^ values. Again, the Q_0_ value was almost equal for both fittings. In this case, the errors in the parameters Q_0_, b, and c_½_ were higher than in the Fe(II) adsorption experiments since the saturation region of the isotherm was not reached. The errors in n and m were small because the slope of the isotherm was well-defined. The adsorption energy of Cr(VI) on GSLP-Fe was lower than the adsorption energy of iron on GSLP. This indicated that Cr(VI) did not displace the adsorbed iron, which is in concordance with the experimental data, where no iron desorption was observed.

#### 3.3.4. Chromium Removal by GSLP-Fe(0)

When GSLP-Fe(0) was used to remove Cr(VI), it was observed that, at the end of the process, Cr(III) appeared in the reaction medium. When GSLP-Fe was used, no Cr(III) appeared at the end of the process. This confirmed that treatment with eucalyptus extract reduced Fe(III) to Fe(0), which was consistent with the properties of the extract described in Section 2.2. Furthermore, after the first hours of reaction, GSLP-Fe(0) lost its characteristic black color and recovered the red color that is characteristic of GSLP-Fe. Therefore, the process that occurred during the treatment with eucalyptus extract was a reversible process. The following reaction is proposed:

(10) $${\mathrm{Cr}}_{2}O_{7}^{2-}{+2\mathrm{Fe}+14H}^{+}\;⇄{\;2\mathrm{Cr}}^{3+}{+2\mathrm{Fe}}^{3+}{+7H}_{2}O$$

This reaction requires a large number of protons, which explains why the largest eliminations occurred at very acidic pH.

When using GSLP-Fe(0) at higher pH, neither Cr(VI) reduction nor Cr(VI) adsorption were observed. However, when GSLP-Fe was used at the same pH, Cr(VI) adsorption was observed. This indicates that Cr(VI) adsorption occurred on Fe(III) but not on Fe(0). Therefore, in the Cr(VI) removal process with GSLP-Fe(0) at acidic pH, the adsorption did not start until Fe(0) was oxidized to Fe(III) in the reduction step.

Figure 9a shows the Cr(VI) removal percentages using GSLP-Fe(0) with respect to the initial Cr(VI) concentration. Figure 9b shows the data in isotherm form without fitting, as this was not a pure adsorption process. One hundred percent removals of Cr(VI) were achieved up to concentrations of 25 mg·L^−1^ using GSLP-Fe(0) with a low material dose (2 g·L^−1^). The maximum Cr(VI) removal values were remarkably high (1.7 g Cr(VI)·g^−1^ Fe). Table 4 shows the removal efficiency of GSLP-Fe(0) compared to other materials and to typical Cr(VI) removers used in industry. Approximately 370 mg was removed by adsorption after 24 h of reaction (Table 3). Therefore, approximately 1.33 g Cr(VI) was removed by reduction, which implies a ratio 2.86–2 between moles of Cr(VI) and moles of Fe(III), while the theoretical ratio according to Equation (10) is 2–2. There are two main reasons for this.

In the Cr(VI) adsorption experiments, the saturation zone of the isotherm was not reached. This caused a high error in the Q_0_ parameter, so the Cr(VI) adsorption could have been higher than 370 mg·g^−1^. In addition, some of the organic matter bound to the iron during the eucalyptus extract may have contributed to the reduction in Cr(VI).

#### 3.3.5. Total Chromium Sorption on GSLP-Fe(0)

As has been shown in the previous sections, adsorption of Cr(VI) on GSLP-Fe(0) was achieved. It has also been shown that, in the Cr(VI) removal process, Cr(III) was generated by reduction. To check if Cr(III) adsorption on GSLP-Fe(0) occurred, experiments were conducted using CrCl_3_. Cr(III) sorption was studied using plain GSLP-Fe(0) and GSLP-Fe(0) with adsorbed Cr(VI). No adsorption of Cr(III) on plain GSLP-Fe(0) was observed, neither with Fe(0) nor after oxidizing the Fe(0) to Fe(III). Cr(III) adsorption was only observed on GSLP-Fe(0) pellets with adsorbed Cr(VI). This indicates that, during the Cr(VI) removal process, since both species were in the reaction medium, both could be adsorbed.

To study the total adsorption of chromium irrespective of its oxidation state, atomic adsorption measurements (FAAS) were conducted. As can be seen in Figure 9b, higher adsorption values than the Cr(VI) adsorption values were obtained. The parameters obtained were similar between the two models, and the value of Q_0_ was quite high. The errors in the parameters were important in the GSLP-Fe(0) D2 fitting because the saturation region of the isotherm was not well-defined.

In the SEM and EDS analyses of GSLP-Fe(0) after the removal process, chromium signals appeared. The intensity of the Cr(VI) signals was higher in the areas with high Fe(III) content. This confirmed that chromium was adsorbed mainly on the areas where Fe(III) was present and not on the silicate.

In similar removal processes, it was found that there was no influence from ionic strength or temperature on the Cr(VI) removal and total chromium adsorption processes [26]. This is a clear advantage when using GSLP-Fe(0) as a chromium remover in industrial processes, as the removal is not affected by the salinity or temperature of the effluent.

Further information, such as SEM images and EDS maps and spectra of GSLP-Fe(0) with adsorbed chromium (Figures S29–S31), can be found in the Supplementary Materials.

## 4. Conclusions

Functionalization of silica-based pellets with Fe(II) and subsequent reduction with a eucalyptus extract yielded the formation of GSLP-Fe(0), which provided particularly satisfactory results as a Cr(VI) remover (1.7 g Cr(VI)·g^−1^ Fe). The reduction of iron retained on the surface of the pellets from treatment with the eucalyptus extract not only improved the performance of the material in chromium removal but also enhanced its resistance against iron stripping back to the solution. The material was easy to produce and handle and removed chromium in a one-pot process.

GSLP-Fe(0) has a nanostructured surface with an average particle diameter of 254 nm. The iron retained in these nanostructures maintains the removal properties of iron nanoparticles but avoids the problems of agglomeration occurring with iron nanoparticles in solution. The iron present on the surface of the silica-based pellets is the only active species participating in the removal of chromium from polluted solutions.

Regarding chromium elimination, the entire removal process is a diffusion-controlled one. The reduction step is governed by intraparticle diffusion, while the adsorption step is mainly governed by film diffusion. One of the great advantages of GSLP-Fe(0) is that there is no need to change the reaction conditions between these two steps. Furthermore, this process does not generate by-products, which are produced using other common decontamination techniques.

## Figures and Tables

**Table 1:** Surface parameters of GSLP-Fe, GSLP-Fe(0), and GSLP-Fe(0) after Cr(VI) desorption and Fe(0) regeneration.

Material	Pellet Diameter	BET Surface (m^2^∙g^−1^)	Pore Volume (cm^3^∙g^−1^)	Average Pore Diameter (nm)
GSLP-Fe	D3	132.11	0.372	11.25
	D2	134.9	0.389	11.55
GSLP-Fe(0)	D3	142.4	0.402	11.30
	D2	148.2	0.415	11.21
GSLP-Fe(0) regenerated	D2	145.4	0.433	11.91

**Table 2:** Kinetic parameters obtained using the diffusion models for Fe(II) sorption and Cr(VI) removal.

Metal	Kinetic Parameters			Webber and Morris Model			Linear Boyd Model	
	Pellet Diameter	c_i_ (mg∙L^−1^)	pH	K_i_ (mg∙g^−1^∙h^−½^)	C (mg∙g^−1^)	Pearson’s r	Intercept	Pearson’s r
Fe(II)	D3	75	Nat	6.2 ± 0.4	2.7 ± 0.5	0.987	6 × 10^−2^ ± 2 × 10^−2^	0.999
	D2	75	Nat	6.8 ± 0.4	4.1 ± 0.5	0.987	4 × 10^−2^ ± 4 × 10^−2^	0.997
Cr(VI) (first linear trend)	D3	50	1.2	6.15 ± 0.10	−1.49 ± 0.11	0.999	−33 × 10^−3^ ± 5 × 10^−3^	0.997
	D3	50	0.8	5.1 ± 0.3	−0.5 ± 0.3	0.987	−8 × 10^−3^ ± 5 × 10^−3^	0.997
	D3	75	0.8	5.8 ± 0.4	−0.5 ± 0.5	0.979	−0.5 × 10^−2^ ± 1.0 × 10^−2^	0.985
	D3	100	0.8	8.3 ± 0.6	−0.8 ± 0.6	0.985	−7 × 10^−2^ ± 5 × 10^−2^	0.972
	D2	50	1.2	5.69 ± 0.11	−1.48 ± 0.13	0.998	−30 × 10^−3^ ± 5 × 10^−3^	0.996
	D2	50	0.8	5.9 ± 0.3	−0.9 ± 0.4	0.988	−28 × 10^−3^ ± 7 × 10^−3^	0.998
	D2	75	0.8	5.1 ± 0.4	−0.6 ± 0.5	0.983	0.9 × 10^−2^ ± 1.6 × 10^−2^	0.989
Cr(VI) (second linear trend)	D3	50	1.2	101 × 10^−2^ ± 3 × 10^−2^	9.21 ± 0.18	0.990	40.2 × 10^−2^ ± 1.3 × 10^−2^	0.999
	D3	50	0.8	106 × 10^−2^ ± 4 × 10^−2^	7.31 ± 0.19	0.991	32.5 × 10^−2^ ± 1.5 × 10^−2^	0.998
	D3	75	0.8	9.6 × 10^−2^ ± 8 × 10^−2^	9.7 ± 0.4	0.989	44 × 10^−2^ ± 3 × 10^−2^	0.993
	D3	100	0.8	9.4 × 10^−2^ ± 4 × 10^−2^	9.6 ± 0.2	0.964	52 × 10^−2^ ± 8 × 10^−2^	0.959
	D2	50	1.2	101 × 10^−2^ ± 3 × 10^−2^	8.23 ± 0.13	0.996	37.2 × 10^−2^ ± 1.1 × 10^−2^	0.999
	D2	50	0.8	190 × 10^−2^ ± 7 × 10^−2^	5.85 ± 0.19	0.996	23 × 10^−2^ ± 3 × 10^−2^	0.997
	D2	75	0.8	2.3 ± 0.2	4.6 ± 0.6	0.978	9 × 10^−2^ ± 9 × 10^−2^	0.966

**Table 3:** Parameters obtained from the isotherm models for Fe(II) sorption, Cr(VI) sorption, and total chromium sorption. The Q_0_ value for Cr(VI) removal was obtained directly from the experimental data without using any isotherm model.

Equilibrium		Langmuir–Freundlich Model				Statistical Thermodynamics Model				
		Q_0_ (mg∙g^−1^)	b (L∙mg^−1^)	n	R^2^	Q_0_ (mg∙g^−1^)	m	c_½_ (mg∙L^−1^)	R^2^	E (kJ∙mol^−1^)
Fe(II) Sorption	D3	13.88 ± 0.10	2.05 ± 0.04	0.24 ± 0.02	0.995	13.88 ± 0.10	4.1 ± 0.3	488×10^−3^ ± 8×10^−3^	0.995	33.17
	D2	16.2 ± 0.4	2.05 ± 0.06	0.15 ± 0.02	0.959	16.2 ± 0.4	6.8 ± 1.0	48.7×10^−2^ ± 1.4×10^−2^	0.959	33.17
Cr(VI) Sorption	D3	370 ± 71	12 × 10^−3^ ± 5 × 10^−3^	1.0 ± 0.2	0.974	370 ± 71	1.0 ± 0.2	87 ± 39	0.974	17.52
	D2	369 ± 71	11 × 10^−3^ ± 4 × 10^−3^	0.8 ± 0.2	0.963	369 ± 71	1.3 ± 0.3	89 ± 35	0.963	17.45
Cr(VI) Removal	D3	1700	-	-	-	1700	-	-	-	-
	D2	1729	-	-	-	1729	-	-	-	-
Total Cr Sorption	D3	898 ± 29	160 × 10^−4^ ± 9 × 10^−4^	0.19 ± 0.04	0.982	898 ± 29	5.0 ± 1.0	63 ± 4	0.982	-
	D2	1371 ± 330	9 × 10^−3^ ± 3 × 10^−3^	0.6 ± 0.3	0.838	1371 ± 329	1.8 ± 1.0	114 ± 44	0.838	-

**Table 4:** Comparison of Cr(VI) removal by GSLP-Fe(0) with other materials and typical Cr(VI) removers in industry.

Cr(VI) Removal (mg·g^−1^)	Material	Reference
1729	GSLP-Fe(0)	Present study
310.6	Wheat bran	[5]
153.8	Chitosan/ceramic alumina Chitosan/perlite	
318	*Penaeus vannamei* prawn shell	[7]
555.6	CS/β-CD/STTP beads	[36]
408.2	SO_2_	[37]
333.8	NaHSO_3_	

## Data Availability

All data that support this work are included in this manuscript and in the Supplementary Material.

## Supplementary Materials

The following supporting information can be downloaded at: https://www.mdpi.com/article/10.3390/nano12223952/s1: Figure S1: Optical images of GSLP D3, GSLP-Fe D3, and GSLP-Fe(0) D3, Figure S2: Optical images of GSLP D2, GSLP-Fe D2, and GSLP-Fe(0) D2, Figure S3: (a,b) SEM images of GSLP (D3). (c–e) EDS maps of (b), Figure S4: SEM and EDS spectra of GSLP D3, Figure S5: (a,b) SEM images of GSLP D2. (c–e) EDS maps of (b), Figure S6: SEM image and EDS spectrum of GSLP D2, Figure S7: (a) SEM image of GSLP-Fe D3. (b–d) EDS maps of (a), Figure S8: SEM image and EDS spectra of GSLP-Fe D3, Figure S9: (a) SEM image of GSLP-Fe D2. (b–d) EDS maps of (a). Figure S10: SEM image and EDS spectra of GSLP-Fe D2, Figure S11: (a) SEM image of GSLP-Fe (0) D3. (b–d) EDS maps of (a), Figure S12: SEM image and EDS spectra of GSLP-Fe(0) D3, Figure S13: SEM image and EDS spectra of GSLP-Fe(0) D2, Figure S14: TGA graphs of GSLP-Fe(0) D2 (a) and GSLP-Fe D2 (b) decomposition in air, Figure S15: IR spectra of the gases emitted during the TGA experiments at temperature of ca. 98 °C, Figure S16: IR spectra of the gases emitted during the TGA experiments at temperature of ca. 260 °C, Figure S17: (a) N_2_ adsorption and desorption isotherms using GSLP-Fe(0) D3. (b) Pore size distribution and cumulative pore volume of GSLP-Fe(0) D3, Figure S18: (a) N_2_ adsorption and desorption isotherms using GSLP-Fe D3. (b) Pore size distribution and cumulative pore volume of GSLP-Fe D3, Figure S19: (a) N_2_ adsorption and desorption isotherms using GSLP-Fe D2. (b) Pore size distribution and cumulative pore volume of GSLP-Fe D2, Figure S20: Optimization of the HCl dose used in the synthesis of GSLP D3 and D2, Figure S21: Kinetic experimental data for Fe adsorption by GSLP D3 and GSLP D2 fitted using Boyd nonlinear model, Figure S22: Representation of q vs. t1/2 used for the Webber–Morris model for Cr(VI) removal using GSLP-Fe(0) D3 (a) and GSLP-Fe(0) D2 (b), Figure S23: Linearized adsorption kinetics for Cr(VI) removal by GSLP-Fe(0) using the Webber–Morris model. (a) First linear part of the kinetics in which the reduction of Cr(VI) to Cr(III) is predominant. (b) Second linear part of the kinetics in which the Cr adsorption is predominant, Figure S24: Experimental data of Fe(II) sorption on GSLP D3 and GSLP D2 fitted with Equation (7), Figure S25: Experimental data obtained for Cr(VI) sorption using GSLP D3 and GSLP D2, Figure S26: (a) SEM image of GSLP (D3) after use as Cr(VI) sorbent. (b) EDS spectrum of (a). (c,d) EDS maps of (a), Figure S27: (a) SEM image of GSLP (D2) after use as Cr(VI) sorbent. (b) EDS spectrum of (a). (c,d) EDS maps of (a), Figure S28: Equilibria experimental data. Solid lines were obtained by fitting to Equation (7), Figure S29: (a) SEM image of GSLP-Fe(0) D3 after use in Cr(VI) removal. (b–d) EDS maps of (a), Figure S30: SEM image and EDS spectra of GSLP-Fe(0) D3 after use in Cr(VI) removal, Figure S31: SEM image and EDS spectra of GSLP-Fe(0) D2 after use in Cr(VI) removal, Table S1. Atomic and weight percentages of the main ions present in the EDS spectra of Figures S4, S6, S8, S10, S12, S13, S26, S27, S30, and S31, Table S2. Kinetic parameters obtained with the Boyd nonlinear model (Equation (2)).
