# Supplementary material for: Towards the Development of Sustainable Hybrid Materials to Achieve High Cr(VI) Removals in a One-Pot Process
Source: Nanomaterials (Basel). 2022 Nov 9;12(22):3952. doi: 10.3390/nano12223952 (PMC9693857; doi:10.3390/nano12223952)
Supplement: Supplementary file 1 [file nanomaterials-12-03952-s001.zip › nanomaterials-2029679-supplementary.pdf]

Supplementary Materials

Towards the Development of Sustainable Hybrid Materials to Achieve High Cr(VI) Removals in a One-Pot Process

David Gómez-Carnota, José L. Barriada * and Roberto Herrero

Departamento de Química and CICA — Centro Interdisciplinar de Química e Bioloxía, Universidade da Coruña, As Carballeiras, s/n, 15071 A Coruña, Spain
* Correspondence: jose.barriada@udc.es; Tel.: +34-881012261

Figures.

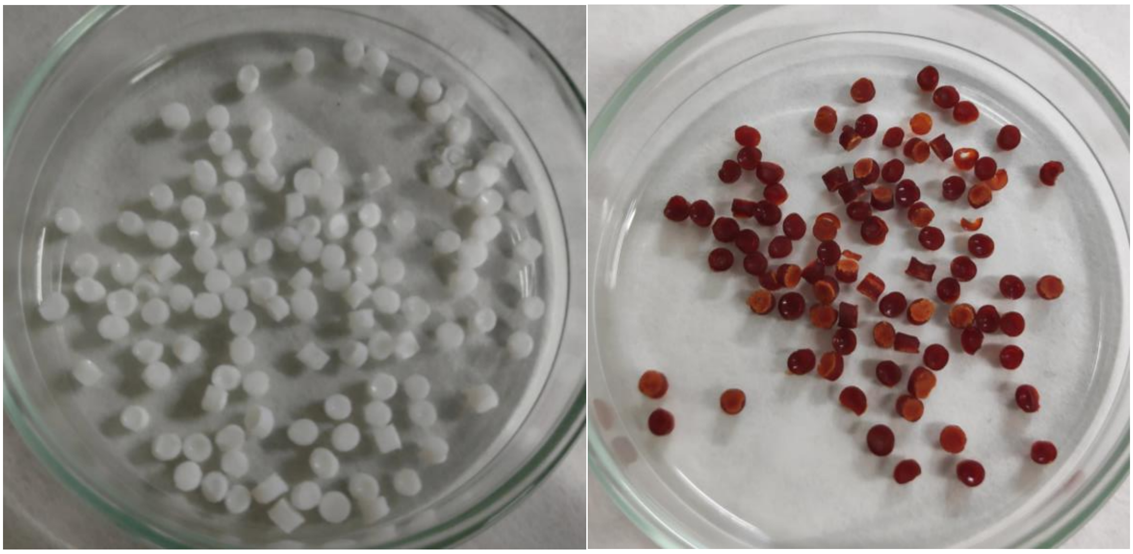

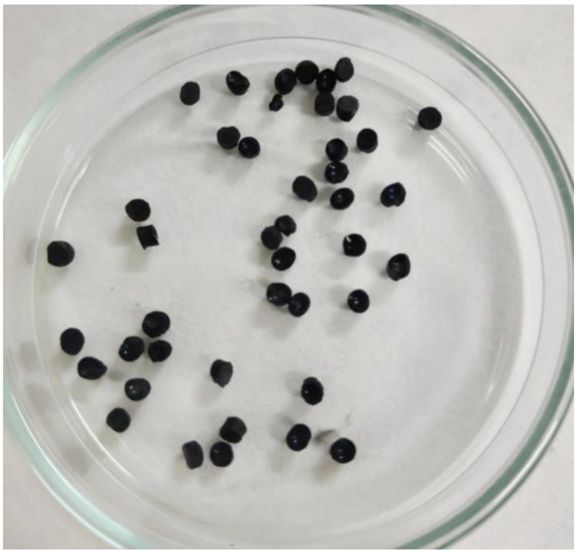

Figure S1. Optical images of GSLP D3 (top, left), GSLP-Fe D3 (top, right) and GSLP-Fe(0) D3 (bottom).

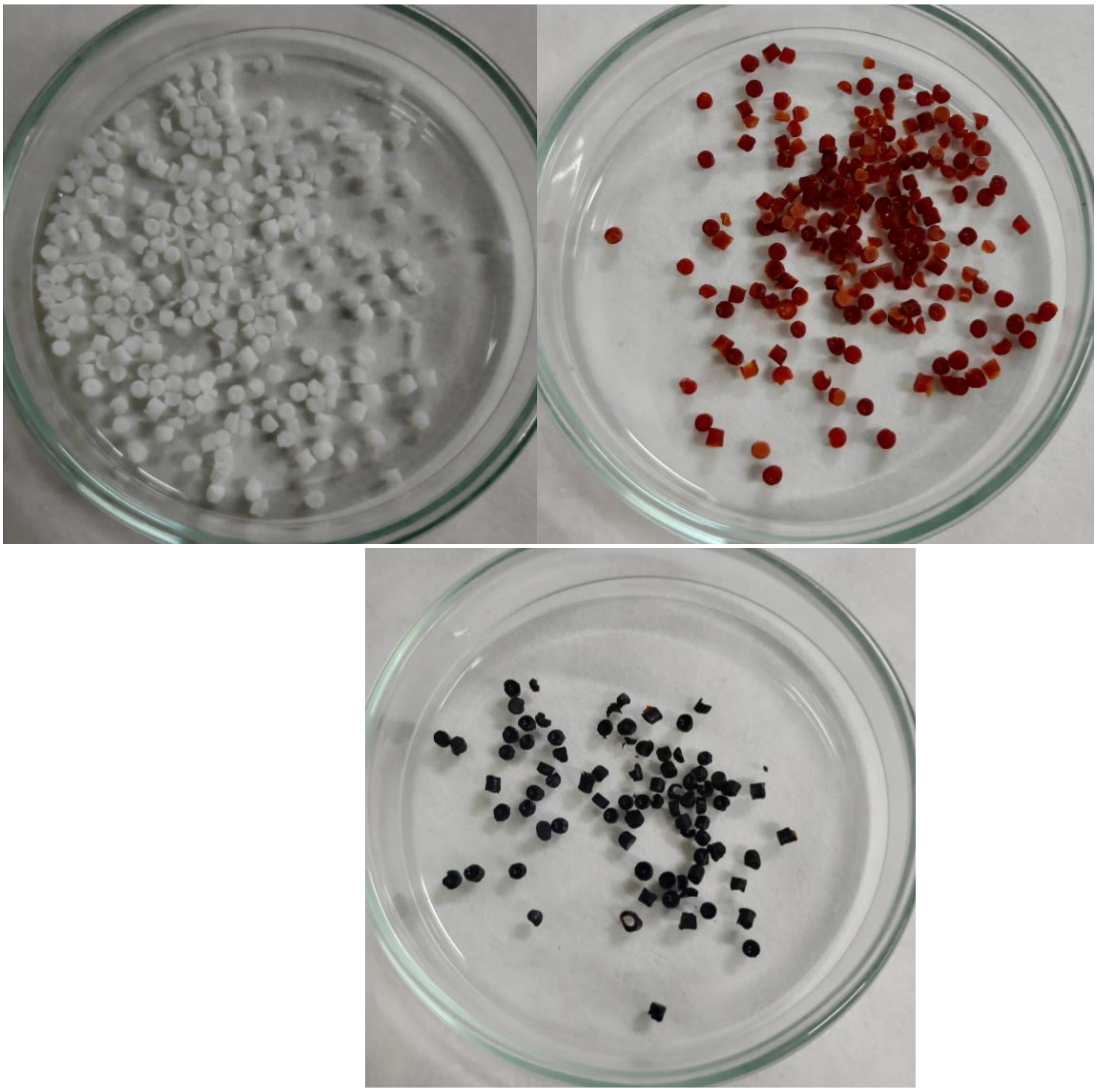

Figure S2. Optical images of GSLP D2 (top, left), GSLP-Fe D2 (top, right) and GSLP-Fe(0) D2 (bottom).

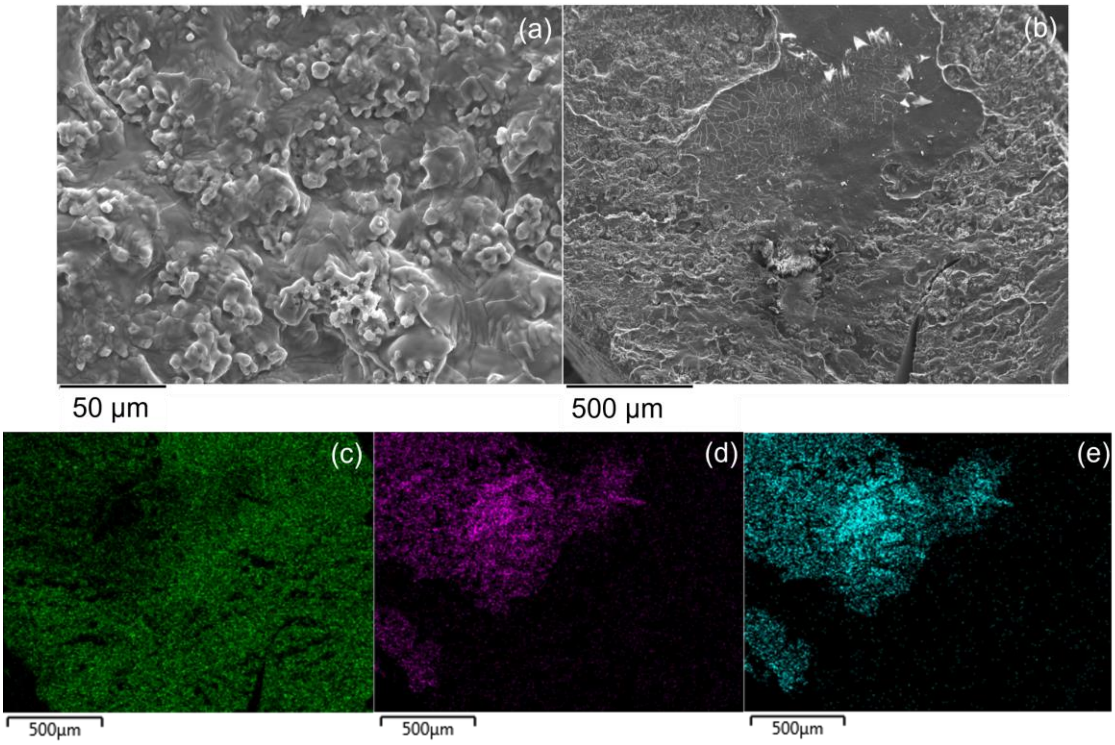

Figure S3. SEM images of GSLP D3. (a) 500x magnification, (b) 60x magnification. EDS maps of (b). Colored zones indicate the presence of each element. Si (c, green), Na (d, purple), Cl (e, cyan).

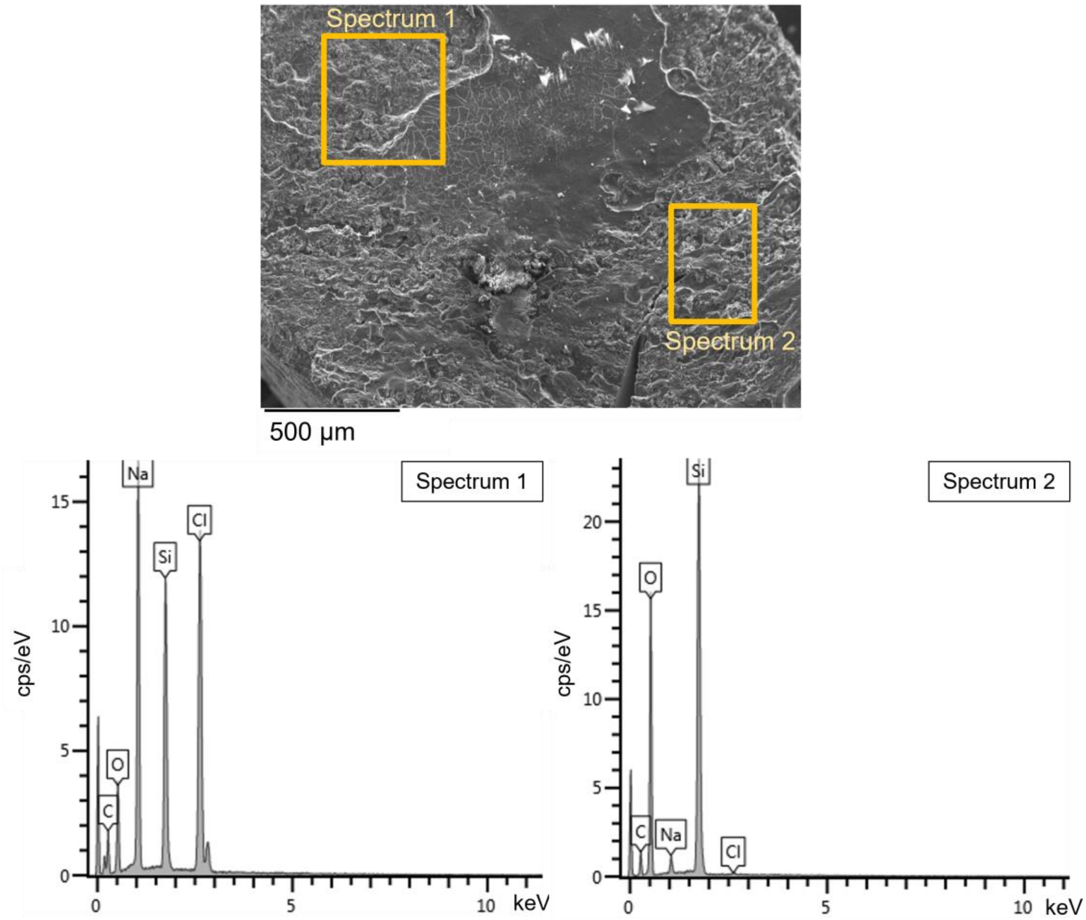

Figure S4. SEM image (60x magnification) and EDS spectra of GSLP D3.

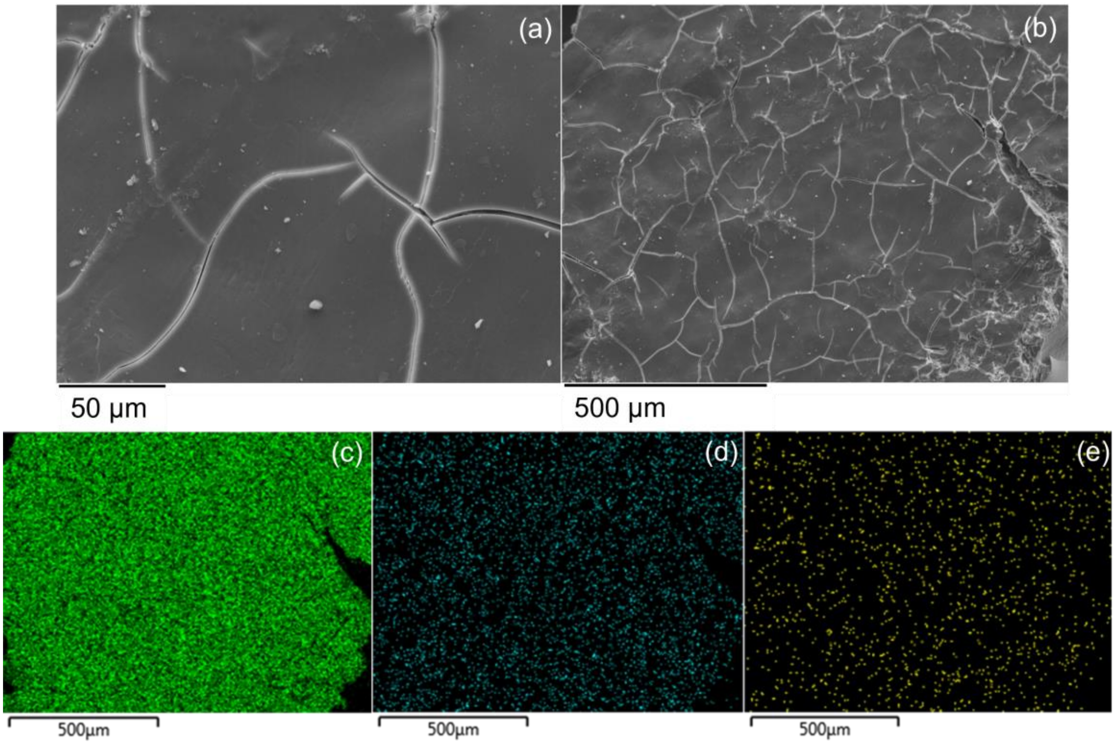

Figure S5. SEM images of GSLP D2. (a) 500x magnification, (b) 95x magnification. EDS maps of (b). Colored zones indicate the presence of each element. Si (c, green), Na (d, cyan), Cl (e, yellow).

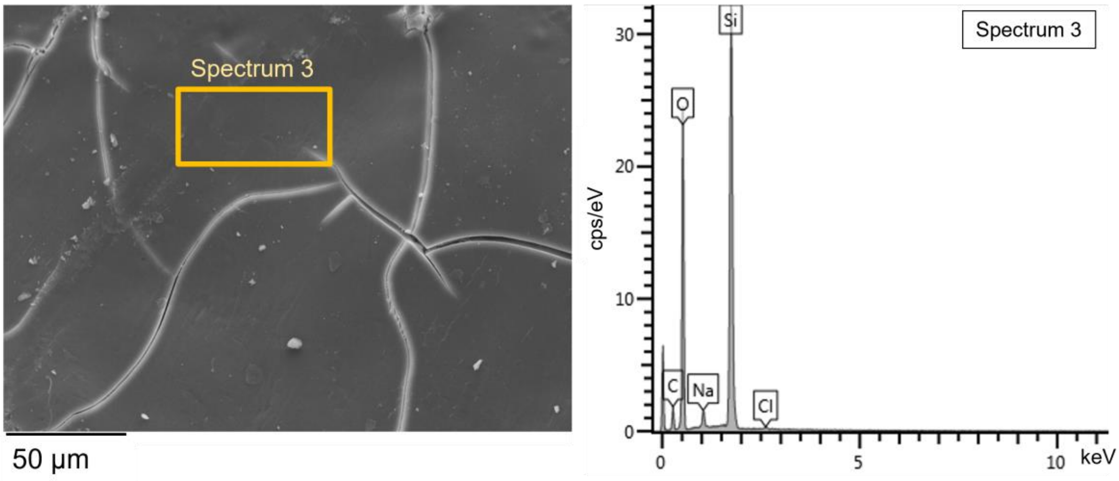

Figure S6. SEM image (500x magnification) and EDS spectrum of GSLP D2.

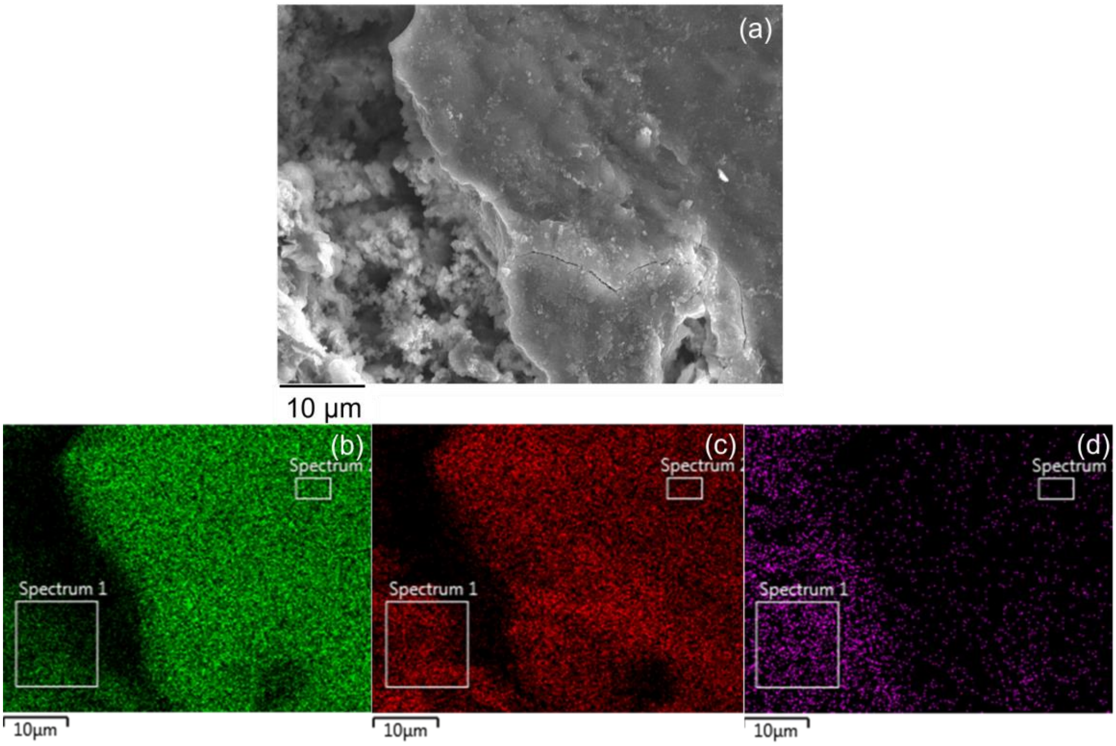

Figure S7. SEM image of GSLP-Fe D3, 2000x magnification (a). EDS maps of (a). Colored zones indicate the presence of each element. Si (b, green), O (c, red), Fe (d, purple).

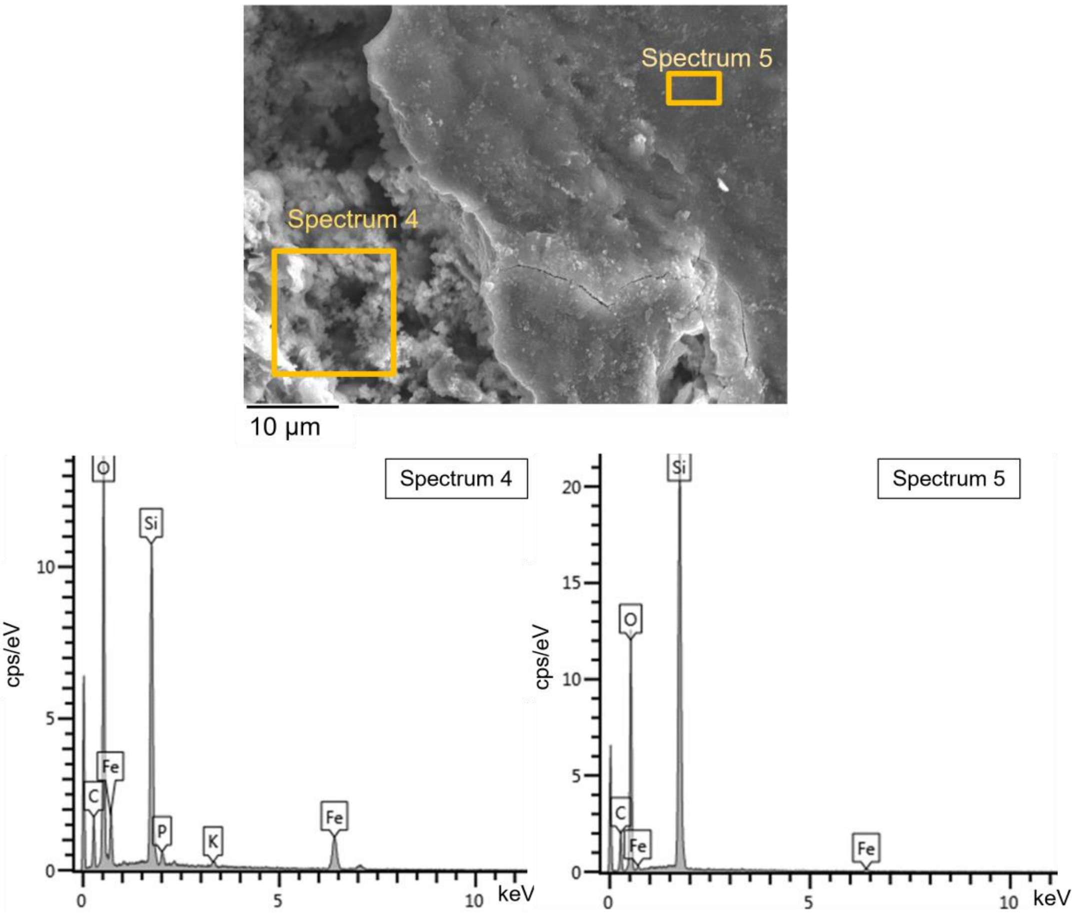

Figure S8. SEM image (2000x magnification) and EDS spectra of GSLP-Fe D3.

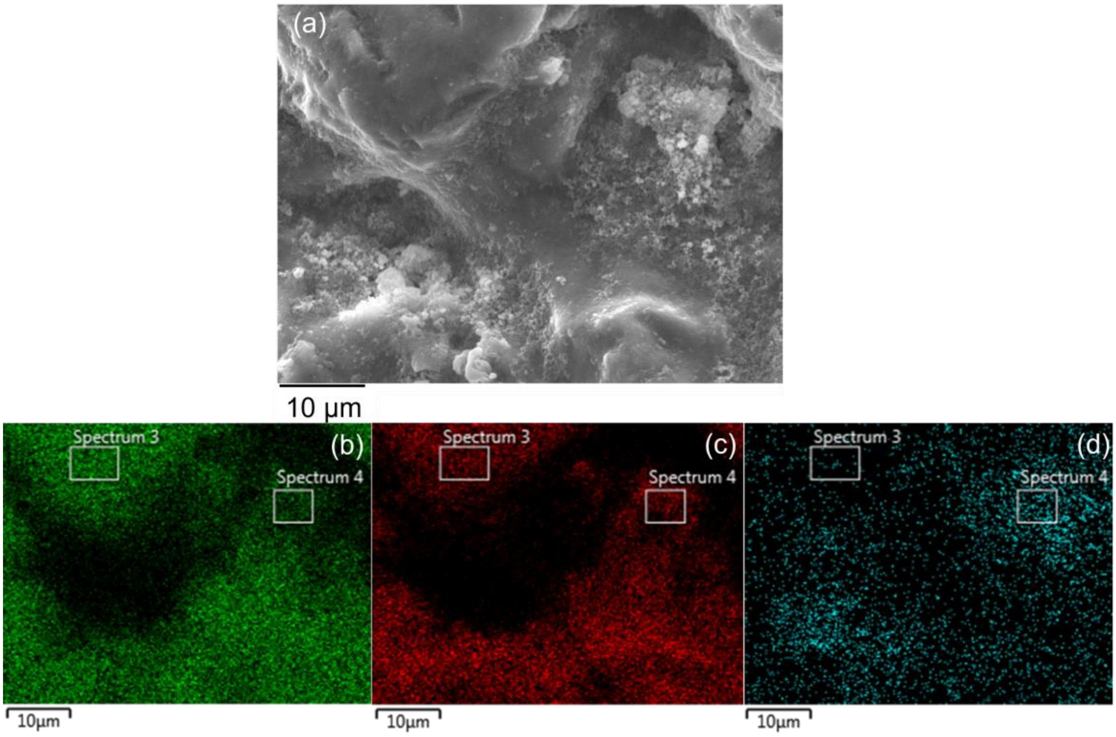

Figure S9. SEM image of GSLP-Fe D2, 2000x magnification (a). EDS maps of (a). Colored zones indicate the presence of each element. Si (b, green), O (c, red), Fe (d, cyan).

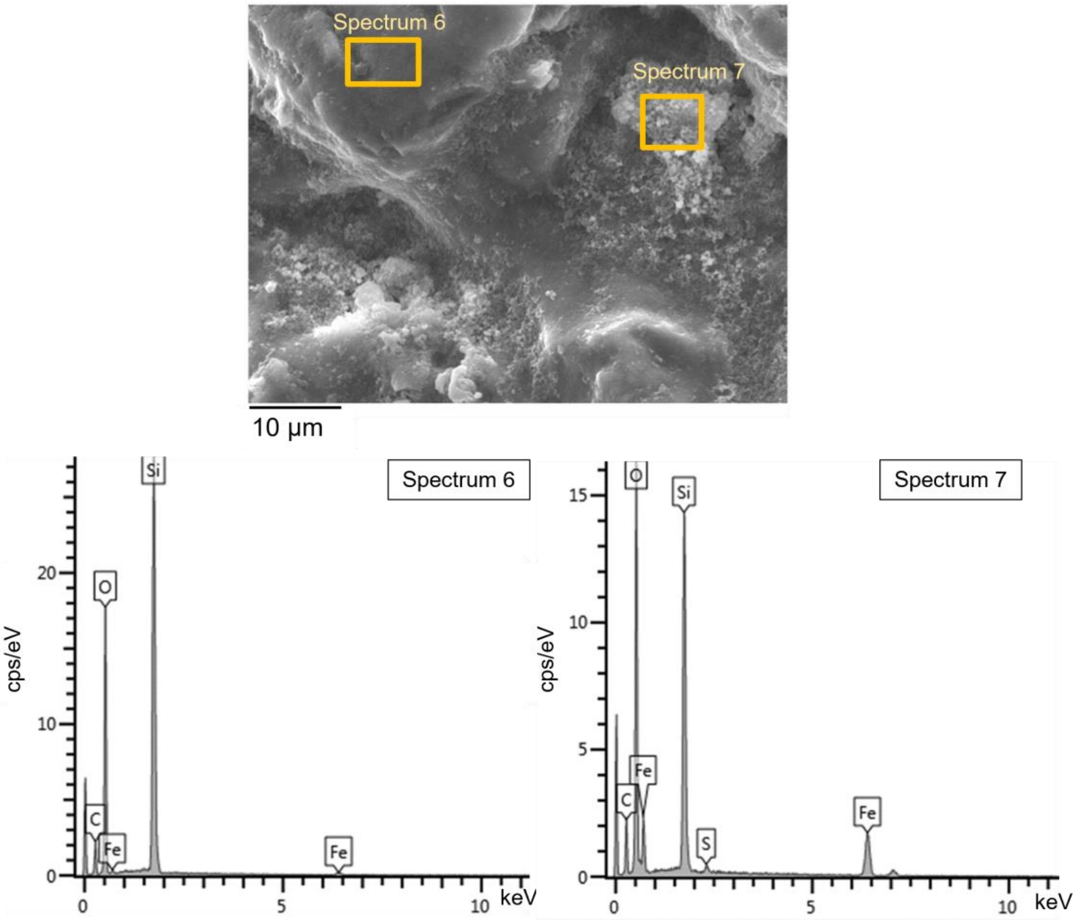

Figure S10. SEM image (2000x magnification) and EDS spectra of GSLP-Fe D2.

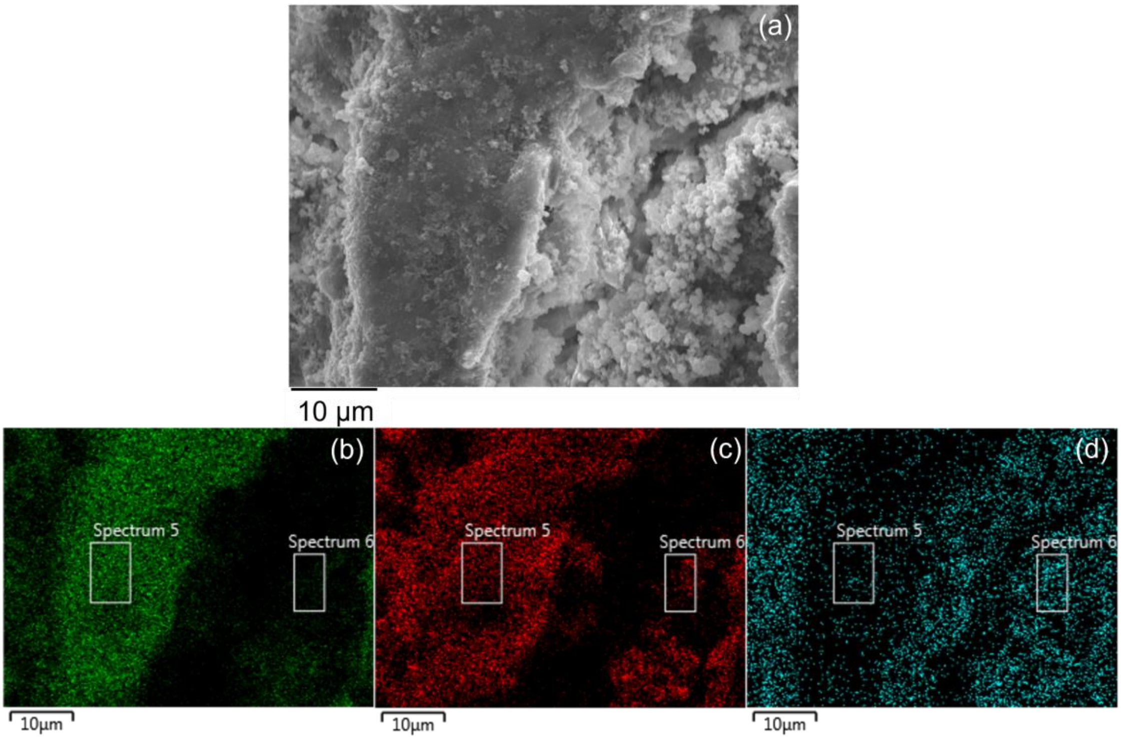

Figure S11. SEM image of GSLP-Fe (0) D3, 2000x magnification (a). EDS maps of (a). Colored zones indicate the presence of each element. Si (b, green), O (c, red), Fe (d, cyan).

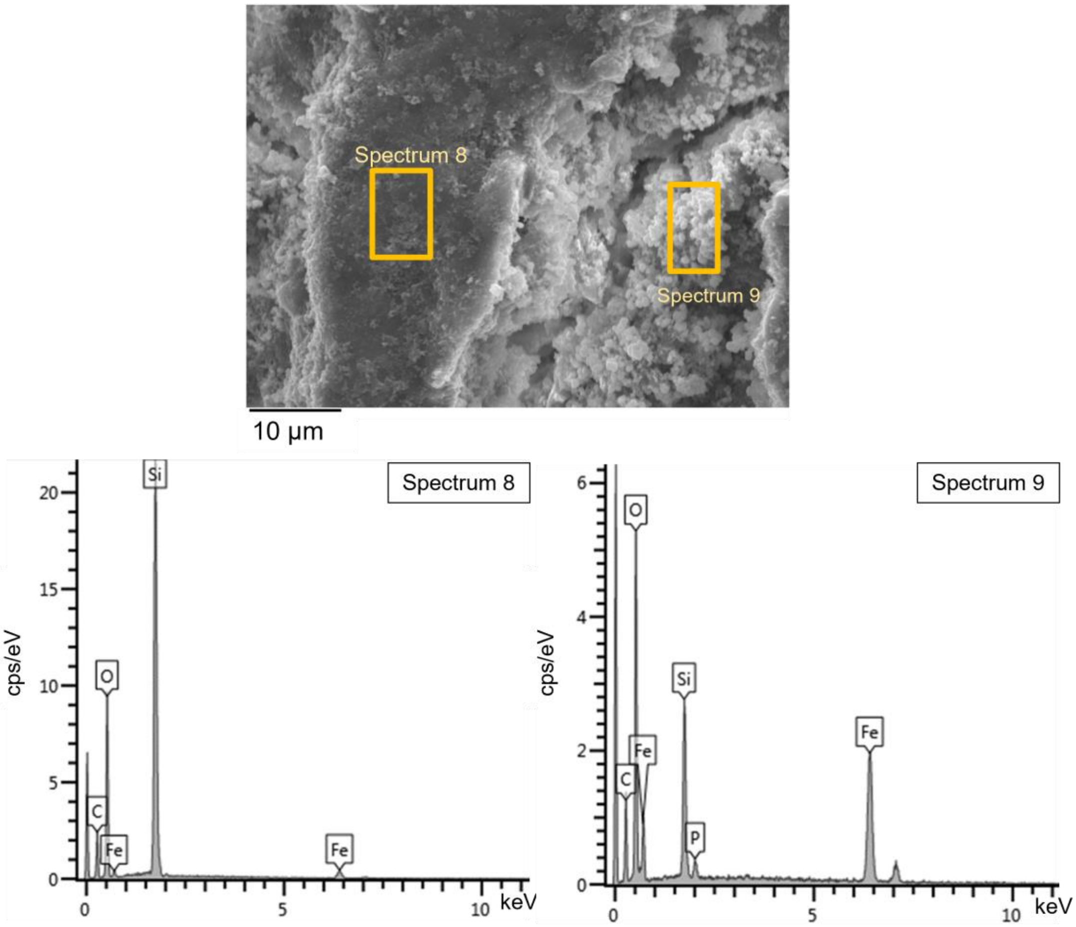

Figure S12. SEM image (2000x magnification) and EDS spectra of GSLP-Fe(0) D3.

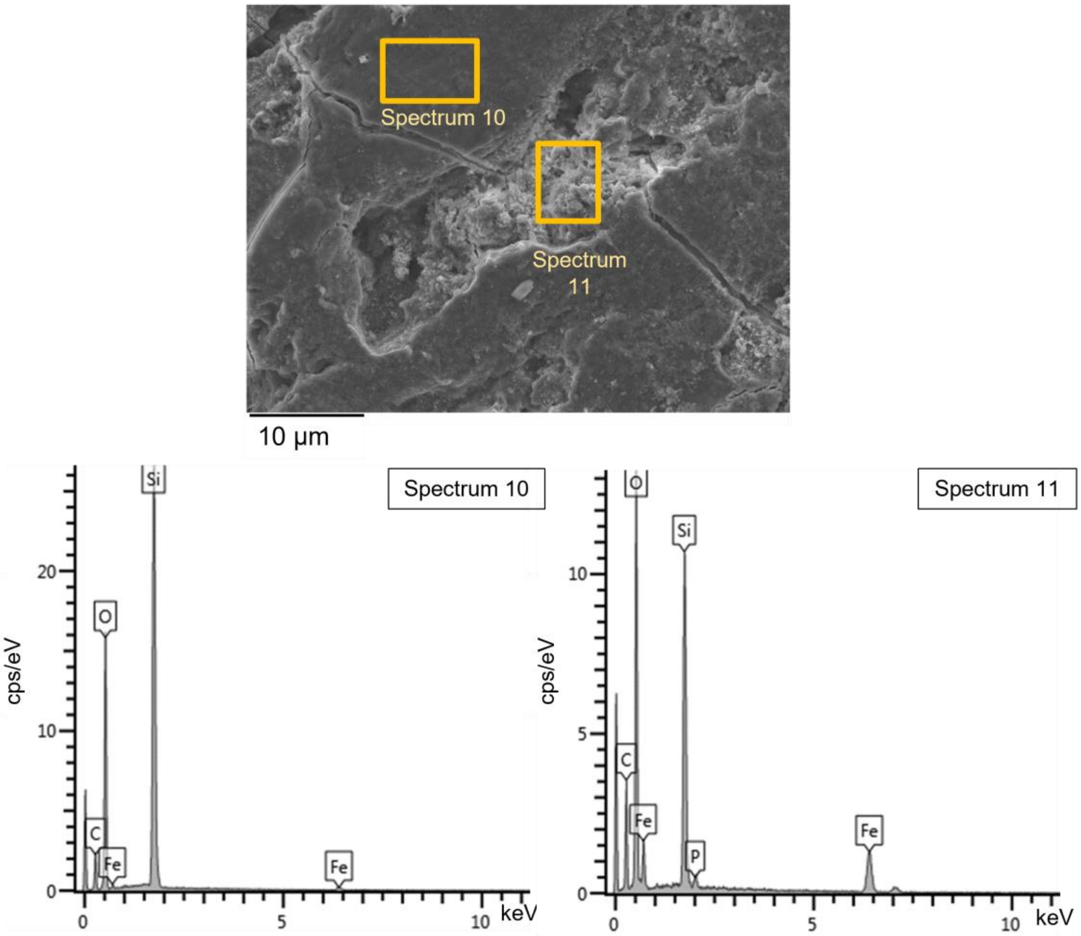

Figure S13. SEM image (500x magnification) and EDS spectra of GSLP-Fe(0) D2.

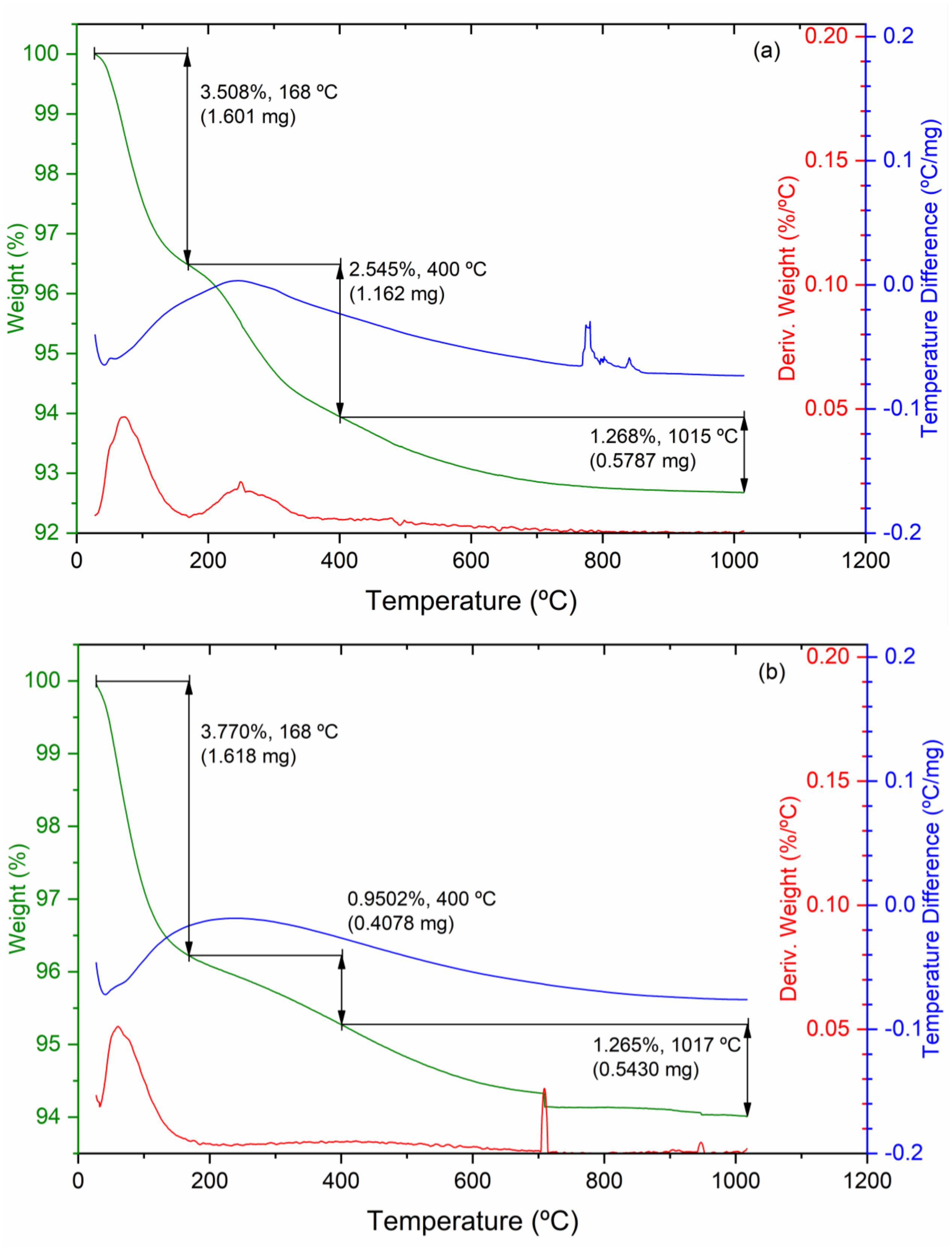

Figure S14. TGA graphs of GSLP-Fe(0) D2 (a) and GSLP-Fe D2 (b) decomposition in air (% Weigh, green). DTG (derivative thermogravimetry, %/°C, red). Temperature difference (°C/mg blue).

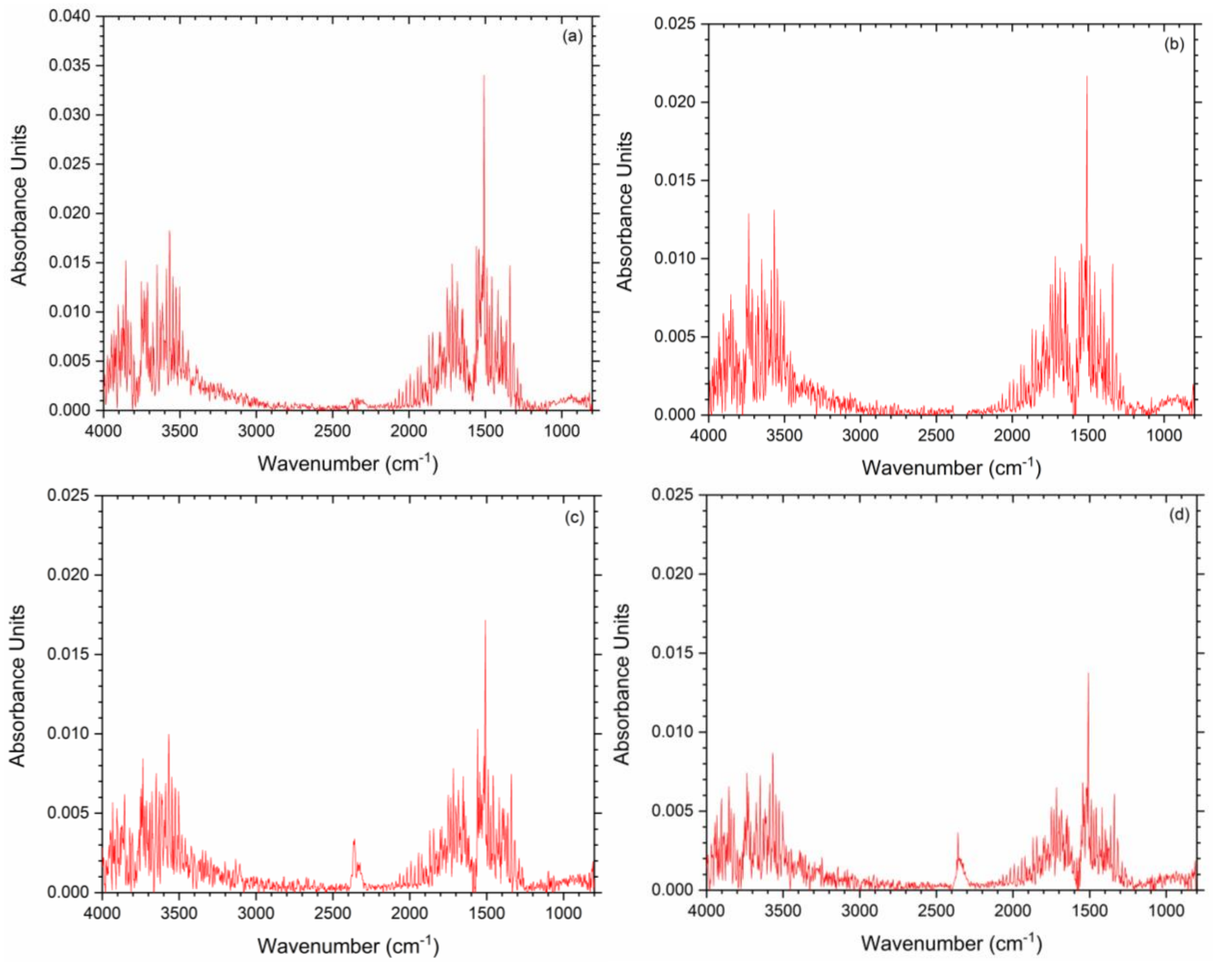

Figure S15. IR spectra of the gases emitted during the TGAs experiments at temperature *ca.* 98 °C. (a) GSLP-Fe(0) D3, 98.5 °C. (b) GSLP-Fe(0) D2, 98.4 °C. (c) GSLP-Fe D3, 97.8 °C. (d) GSLP-Fe D2, 98.3 °C.

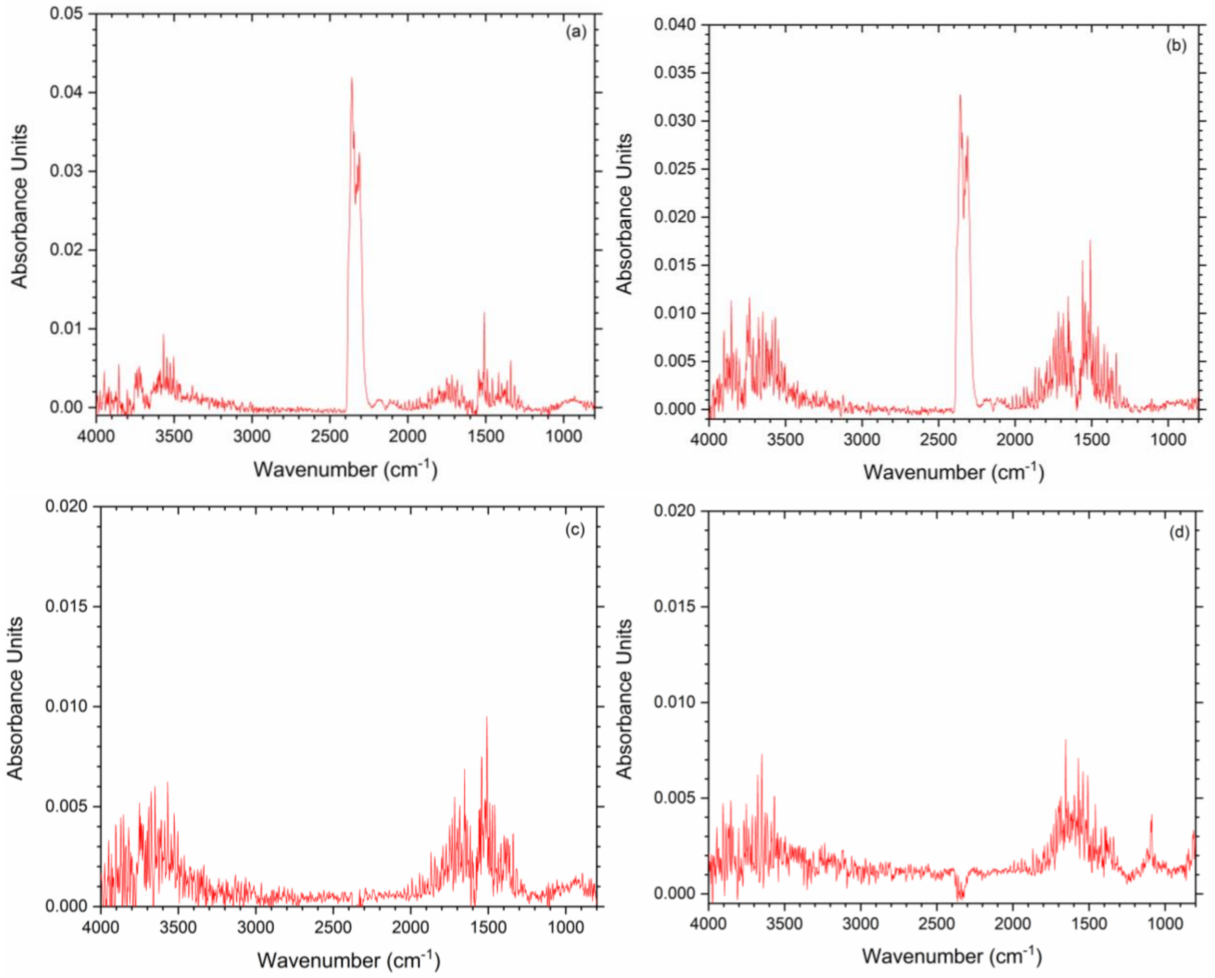

Figure S16. IR spectra of the gases emitted during the TGAs experiments at temperature *ca.* 260 °C. (a) GSLP-Fe(0) D3, 256.7 °C. (b) GSLP-Fe(0) D2, 262.1 °C. (c) GSLP-Fe D3, 260.5 °C. (d) GSLP-Fe D2, 261.2 °C.

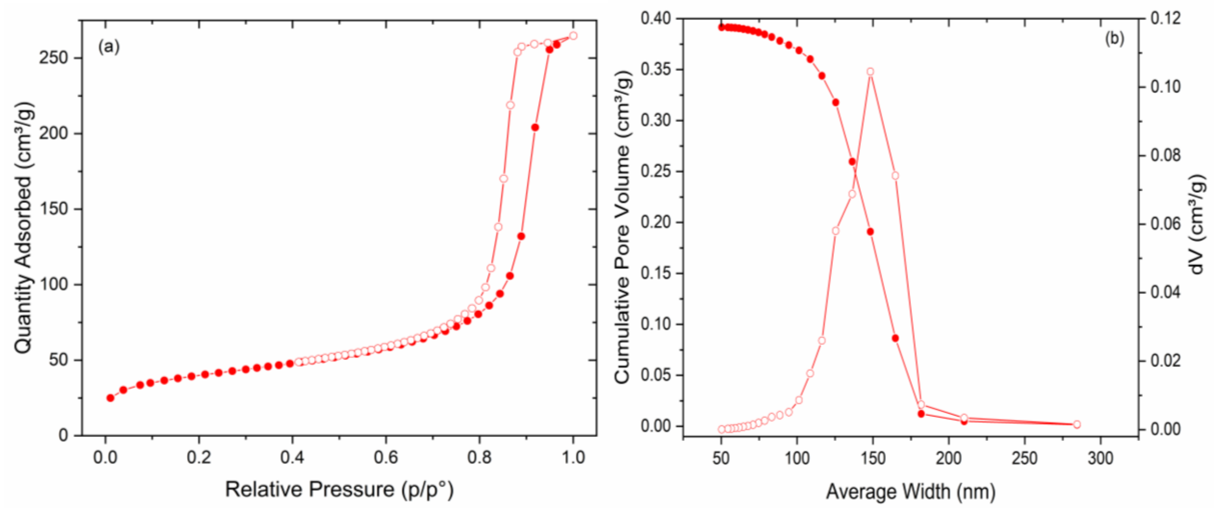

Figure S17. (a) N_2 adsorption (filled circles) and desorption (empty circles) isotherm using GSLP-Fe(0) D3. T = 77 K. (b) Pore size distribution (hollow circles) and cumulative pore volume (filled circles) of GSLP-Fe(0) D3.

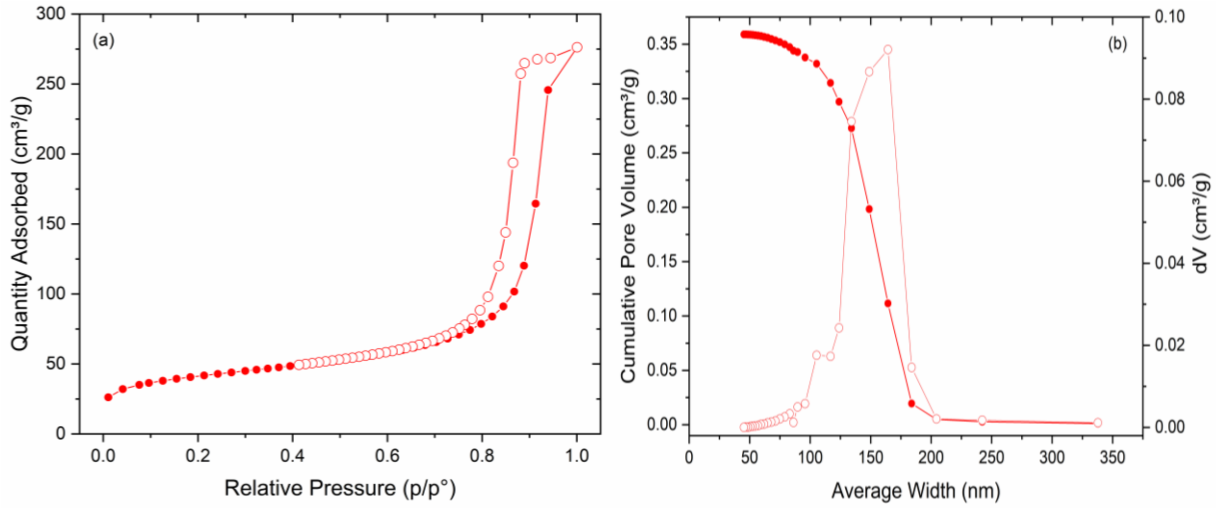

Figure S18. (a) N_2 adsorption (filled circles) and desorption (empty circles) isotherm using GSLP-Fe D3. T = 77 K. (b) Pore size distribution (hollow circles) and cumulative pore volume (filled circles) of GSLP-Fe D3.

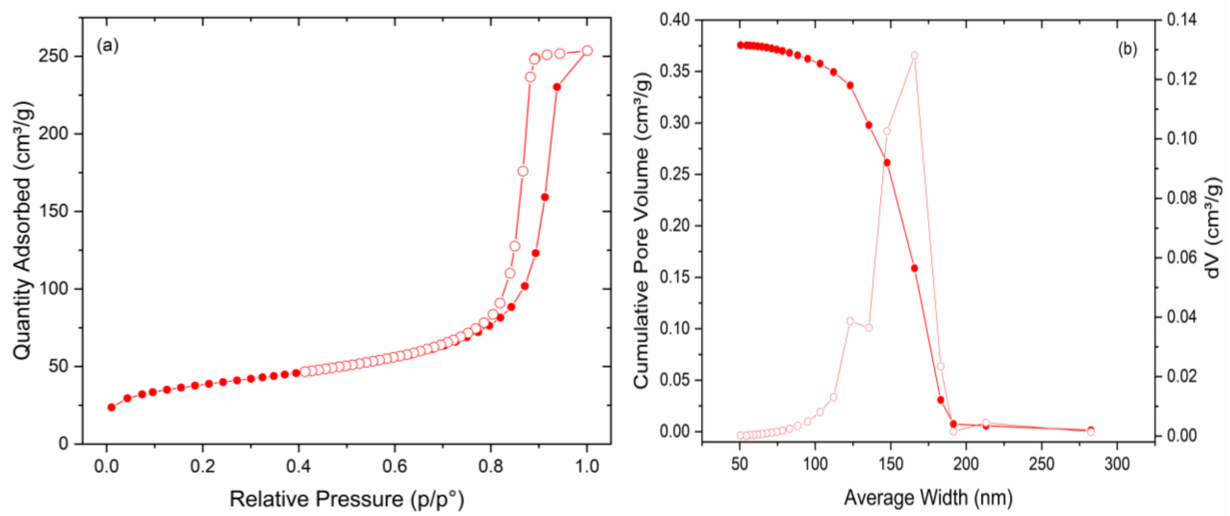

Figure S19. (a) N_2 adsorption (filled circles) and desorption (empty circles) isotherm using GSLP-Fe D2. T = 77 K. (b) Pore size distribution (hollow circles) and cumulative pore volume (filled circles) of GSLP-Fe D2.

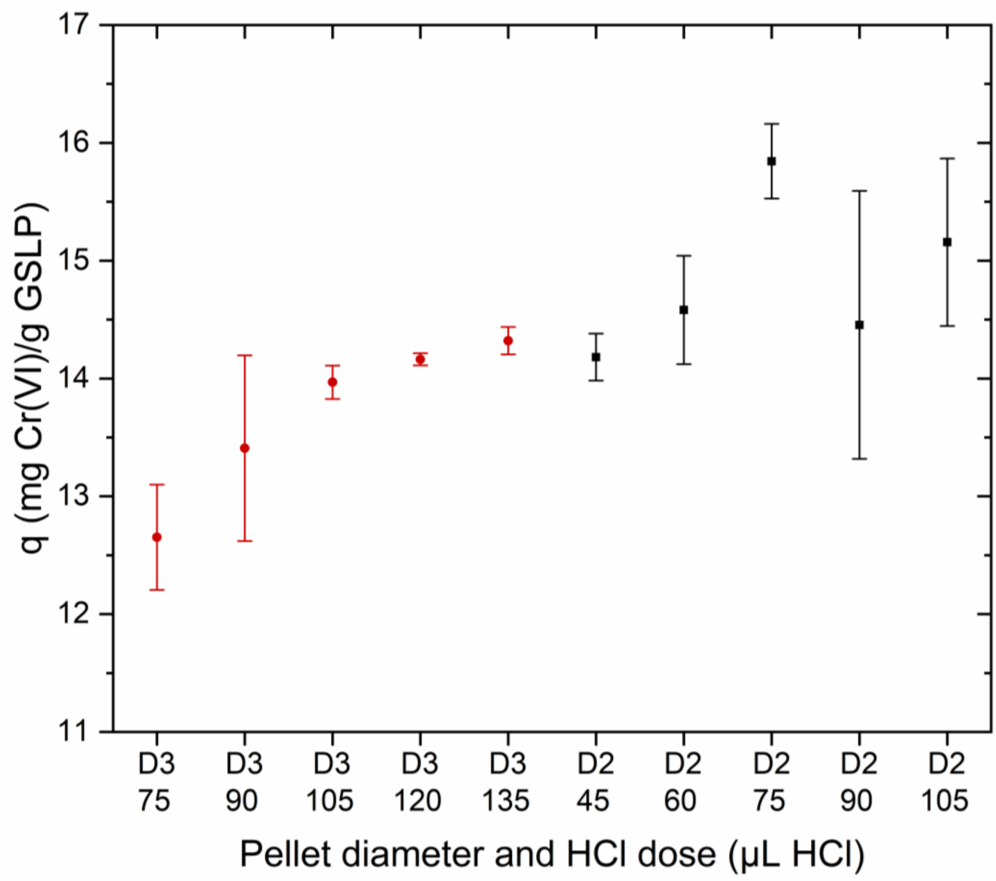

Figure S20. Optimization of the HCl dose used in the synthesis of GSLP pellets D3(●, red) and D2 (■, black). Na_2SiO_3 dose for the synthesis 3 ml. Ludox dose for the synthesis 3 ml. q values represent the amount of Cr(VI) removed after the GSLP transformations with iron and eucalyptus extract, referred to the weight of the initial GSLP.

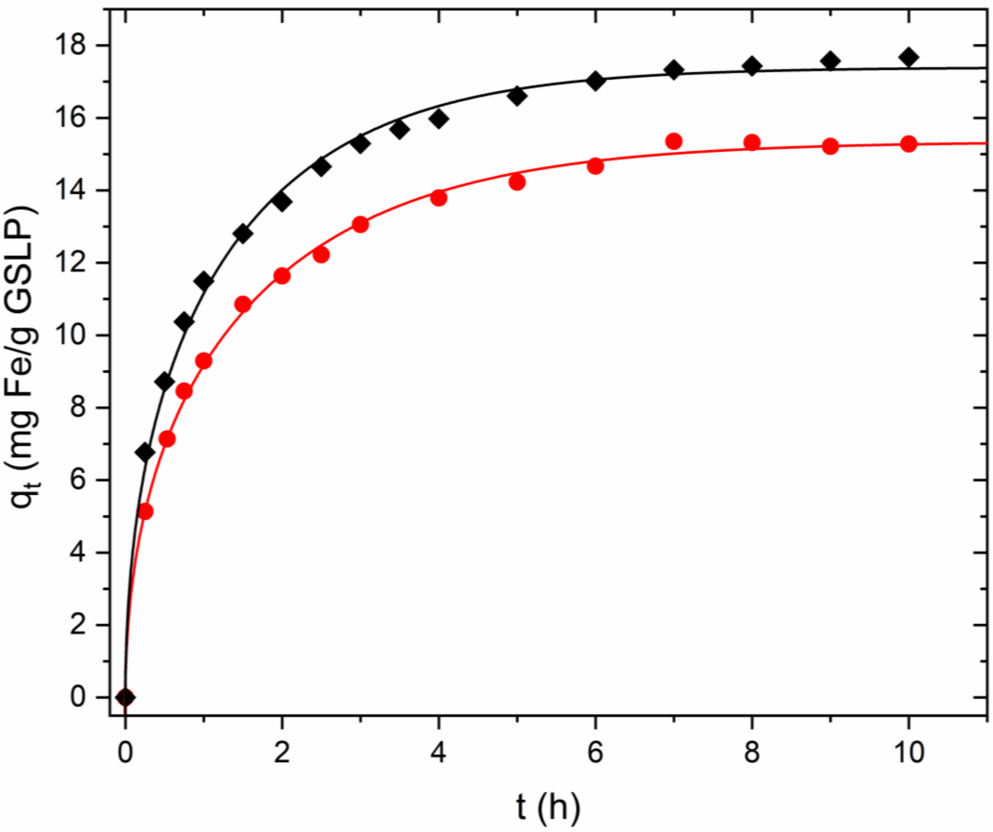

Figure S21. Kinetic experimental data of Fe adsorption by GSLP D3 (●) and GSLP D2 (◆) fitted using Boyd non-linear model. GSLP dose 2 g·L⁻¹, natural pH (*ca.* 5), room temperature, and stirring at 200 RPM. Initial Fe(II) concentration 75 mg·L⁻¹.

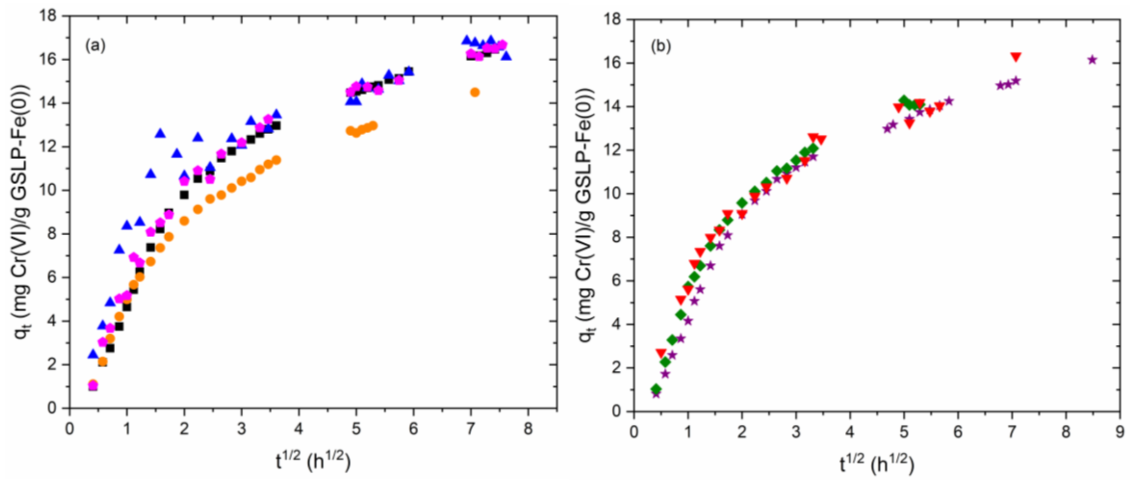

Figure S22. Representation of q vs t1/2 used for the Webber-Morris model for Cr(VI) removal using GSLP-Fe(0) D3 (a) and GSLP-Fe(0) D2 (b). Two distinct linear trends appear in each representation. These two trends correspond to each of the two processes of Cr(VI) removal. First, reduction of Cr(VI) to Cr(III). Second, adsorption of Cr on the material.
GSLP-Fe(0) D3 dose 2 g·L⁻¹, equals to 27.8 mg·L⁻¹ of Fe, GSLP-Fe(0) D2 dose 2 g·L⁻¹, equals to 32.5 mg·L⁻¹ of Fe, room temperature and stirring at 200 RPM for all experiments. (■) HCl 4M dose 1 ml (pH ≈ 1.2), initial Cr(VI) concentration 50 mg·L⁻¹, (●) HCl 4M dose 2 ml (pH ≈ 0.8), Initial Cr(VI) concentration 50 mg·L⁻¹, (⬟) HCl 4M dose 2 ml (pH ≈ 0.8), initial Cr(VI) concentration 75 mg·L⁻¹, (▲) HCl 4M dose 2 ml (pH ≈ 0.8), initial Cr(VI) concentration 100 mg·L⁻¹. (★) HCl 4M dose 1 ml (pH ≈ 1.2), initial Cr(VI) concentration 50 mg·L⁻¹, (◆) HCl 4M dose 2 ml (pH ≈ 0.8), Initial Cr(VI) concentration 50 mg·L⁻¹, (▼) HCl 4M dose 2 ml (pH ≈ 0.8), initial Cr(VI) concentration 75 mg·L⁻¹.

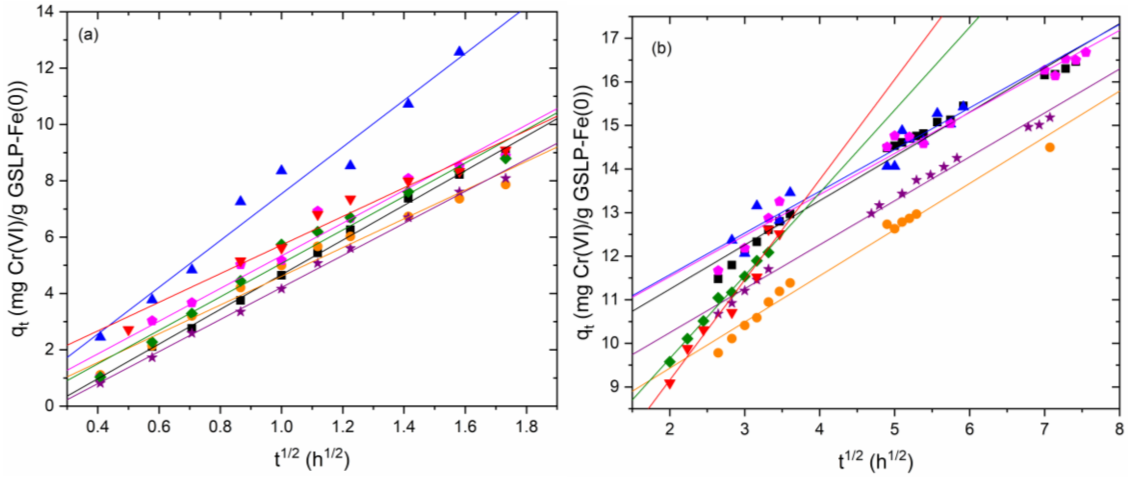

Figure S23. Linearized adsorption kinetics of Cr(VI) removal by GSLP-Fe(0) using the Webber-Morris model. (a) First linear part of the kinetics in which the reduction of Cr(VI) to Cr(III) is predominant. (b) Second linear part of the kinetics in which the Cr adsorption is predominant. (■) HCl 4M dose 1 ml (pH ≈ 1.2), initial Cr(VI) concentration 50 mg·L⁻¹, (●) HCl 4M dose 2 ml (pH ≈ 0.8), Initial Cr(VI) concentration 50 mg·L⁻¹, (⬟) HCl 4M dose 2 ml (pH ≈ 0.8), initial Cr(VI) concentration 75 mg·L⁻¹, (▲) HCl 4M dose 2 ml (pH ≈ 0.8), initial Cr(VI) concentration 100 mg·L⁻¹. (★) HCl 4M dose 1 ml (pH ≈ 1.2), initial Cr(VI) concentration 50 mg·L⁻¹, (◆) HCl 4M dose 2 ml (pH ≈ 0.8), Initial Cr(VI) concentration 50 mg·L⁻¹, (▼) HCl 4M dose 2 ml (pH ≈ 0.8), initial Cr(VI) concentration 75 mg·L⁻¹.

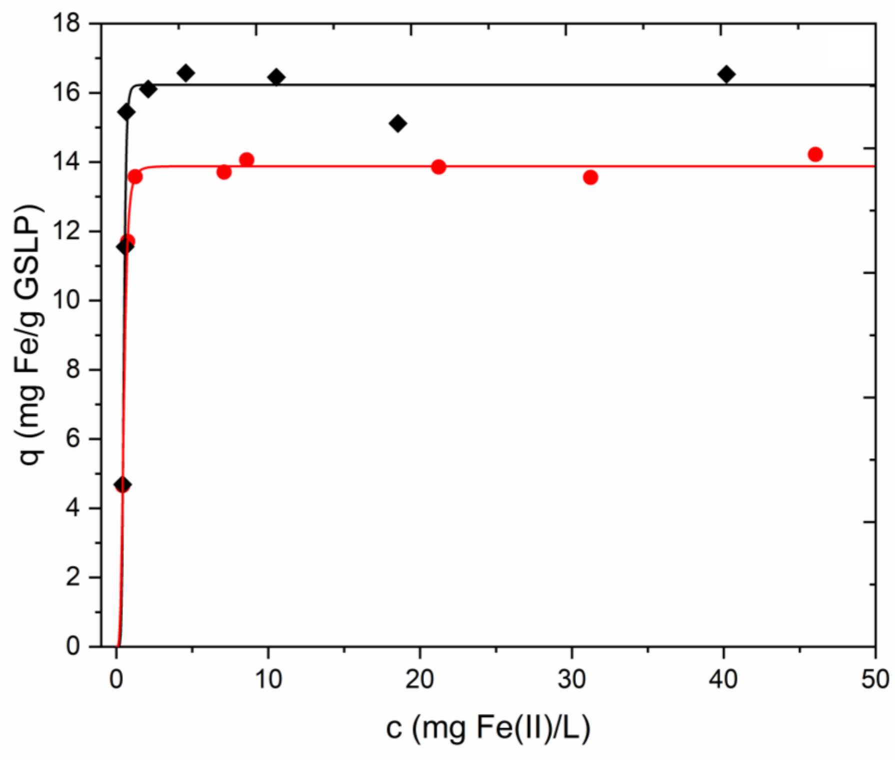

Figure S24. Experimental data of Fe(II) sorption on GSLP D3 (●) and GSLP D2 (◆) fitted with Eq. (7). GSLP dose 2 g·L⁻¹, natural pH (*ca.* 5), room temperature, and stirring at 200 RPM. Initial Fe(II) concentration from 10 to 75 mg·L⁻¹.

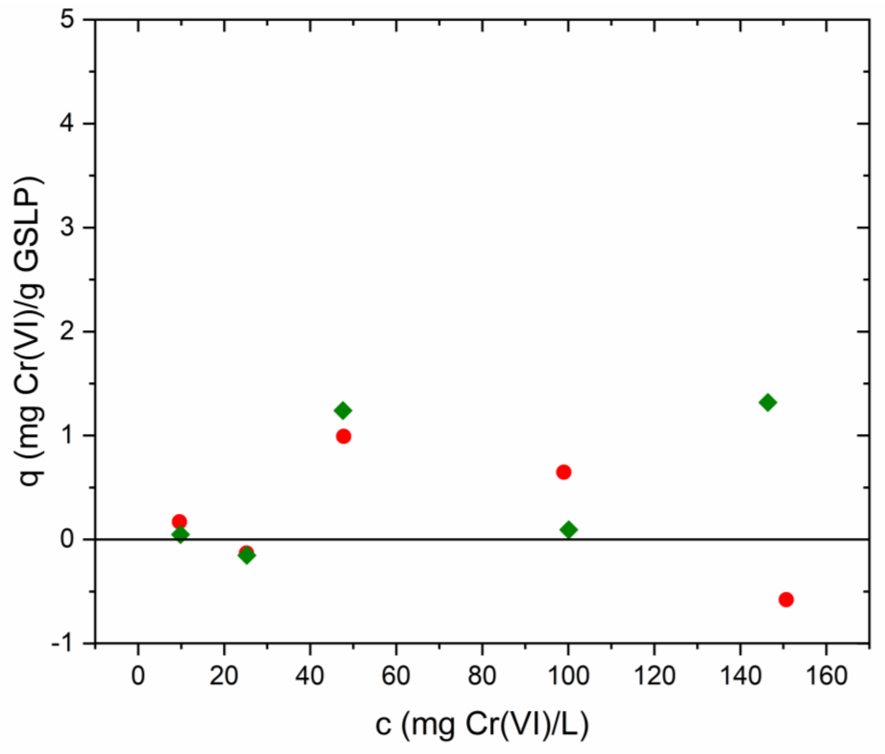

Figure S25. Experimental data obtained for Cr(VI) sorption using GSLP D3 (●) and GSLP D2 (◆). GSLP dose 2 g·L^{-1}, room temperature, pH 0.8 and stirring at 200 RPM for all experiments.

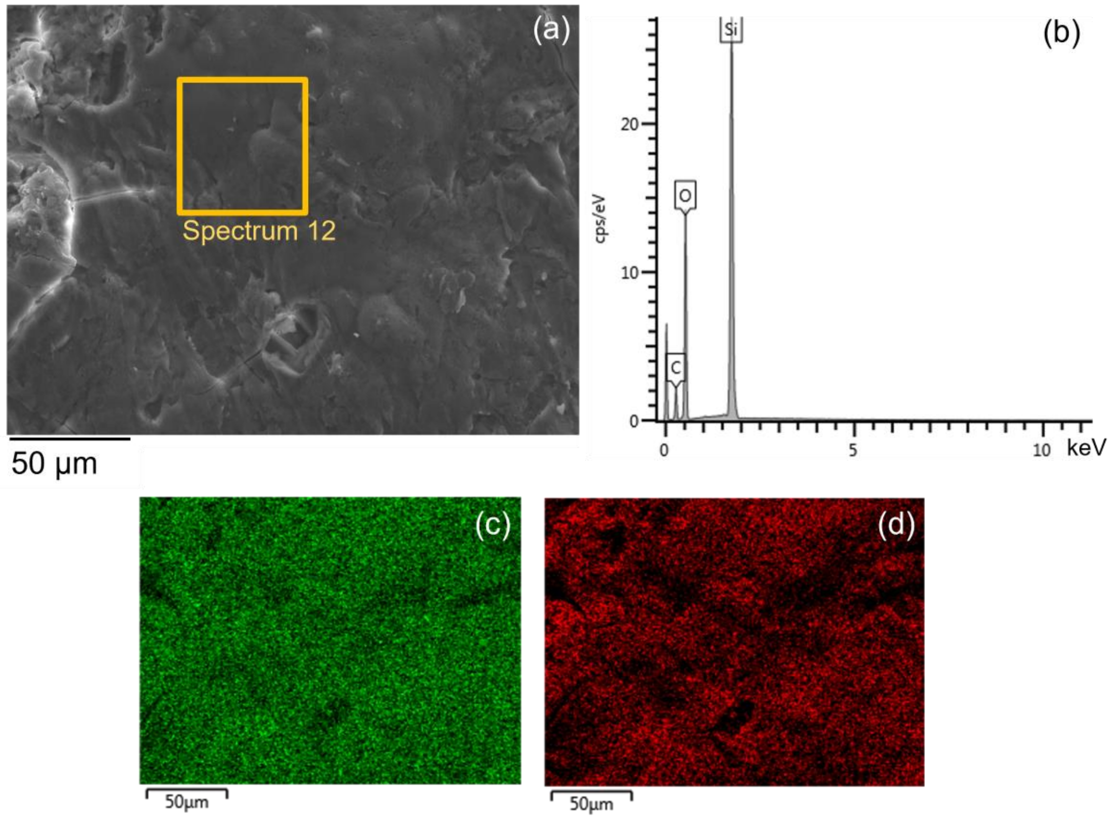

Figure S26. (a) SEM image of GSLP (D3) after use it as Cr(VI) sorbent, 500x magnification. (b) EDS spectrum of (a). (c, d) EDS maps of (a). Colored zones indicate the presence of each element. Si (b, green), O (c, red). No chromium signal is observed.

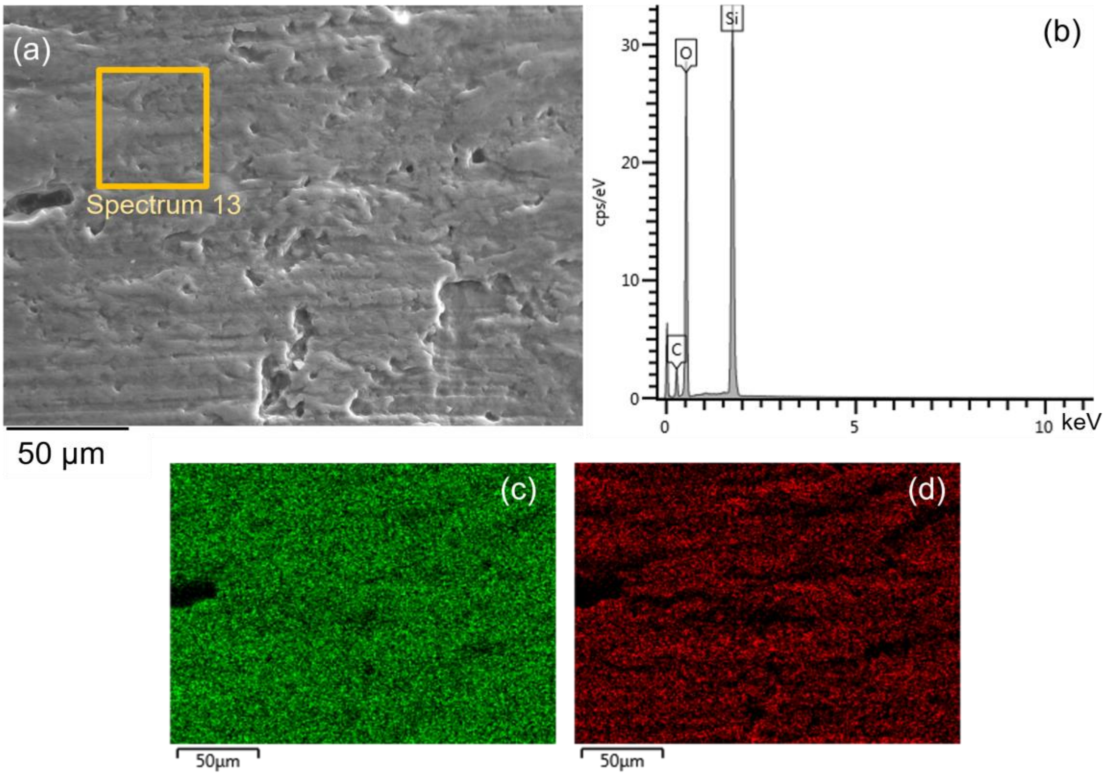

Figure S27. (a) SEM image of GSLP (D2) after use it as Cr(VI) sorbent, 500x magnification. (b) EDS spectrum of (a). (c. d) EDS maps of (a). Colored zones indicate the presence of each element. Si (b, green), O (c, red). No chromium signal is observed.

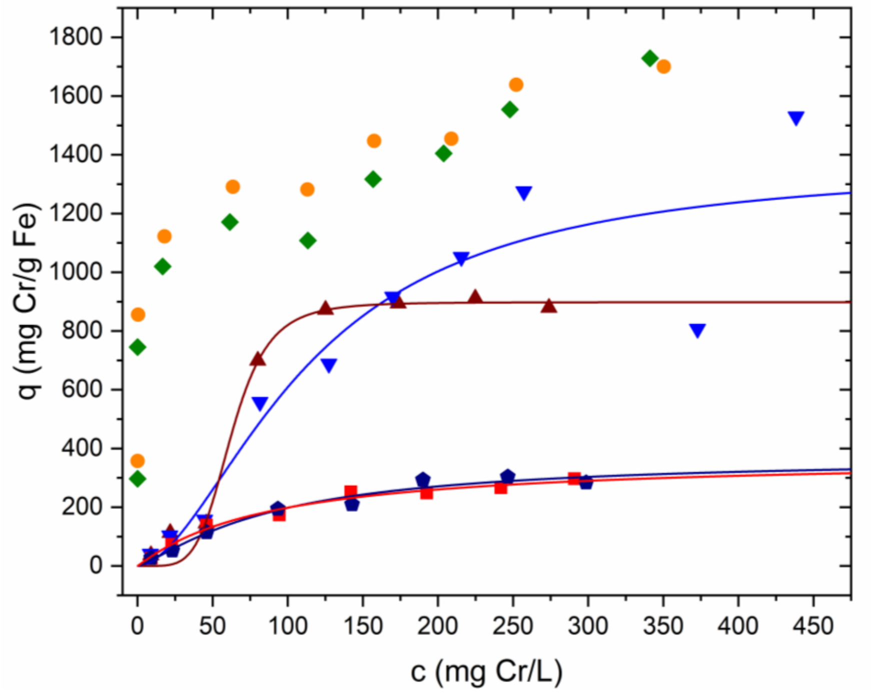

Figure S28. Equilibria experimental data. Solid lines were obtained by fitting to Eq. (7). (■) Cr(VI) adsorption by GSLP-Fe D3. (⬟) Cr(VI) adsorption by GSLP-Fe D2. (●) Cr(VI) maximum elimination by GSLP-Fe(0) D3. (♦) Cr(VI) maximum elimination by GSLP-Fe(0) D2. (▲) Total chromium adsorption by GSLP-Fe(0) D3. (▼) Total chromium adsorption by GSLP-Fe(0) D2. GSLP-Fe/Fe(0) D3 dose 2 $g\cdot L^{-1}$ equals to 27.8 $mg\cdot L^{-1}$ of Fe. GSLP-Fe/Fe(0) D2 dose 2 $g\cdot L^{-1}$ equals to 32.5 $mg\cdot L^{-1}$ of Fe, pH 1, room temperature and stirring at 200 RPM for all the experiments. Initial Cr(VI) concentration from 10 to 500 $mg\cdot L^{-1}$.

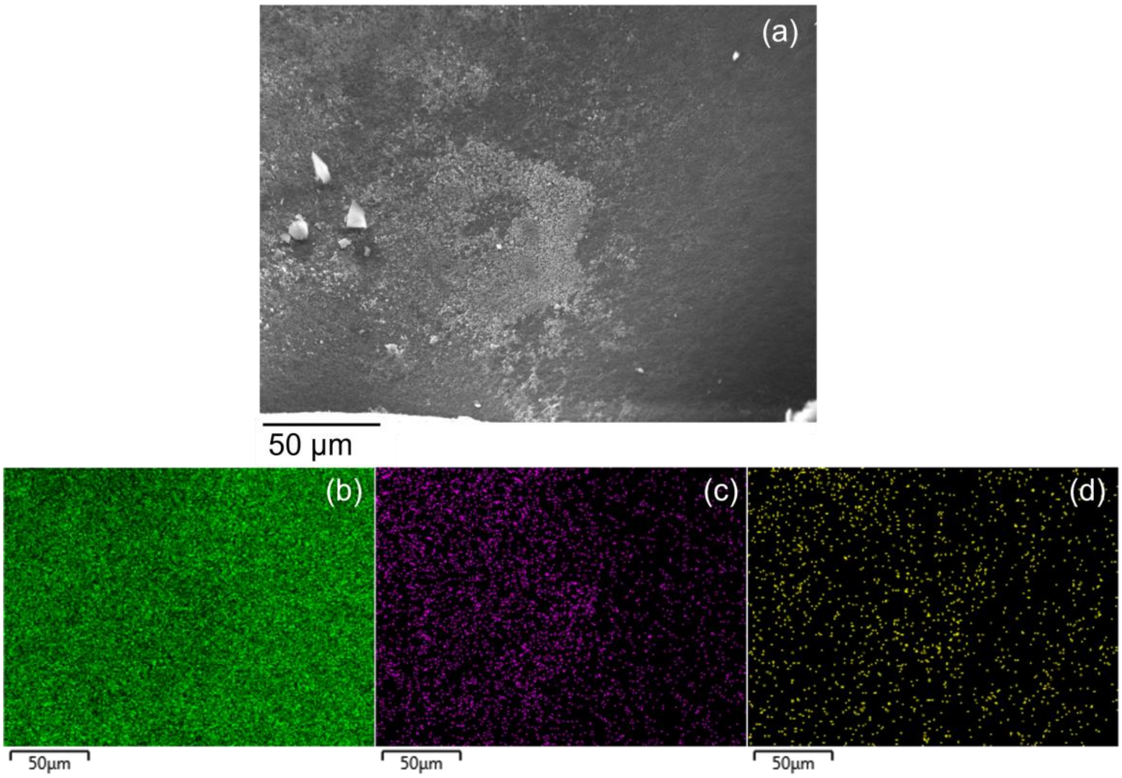

Figure S29. SEM image of GSLP-Fe(0) D3 after use it in Cr(VI) removal, 500x magnification (same as the blanks, Fig. SFigure **S26** and SFigure **S27**) (a). EDS maps of a. Colored zones indicate the presence of each element. Si (b, green), Fe (c, purple), Cr (d, yellow). The density of yellow points is higher in the area where the density of purple points is higher.

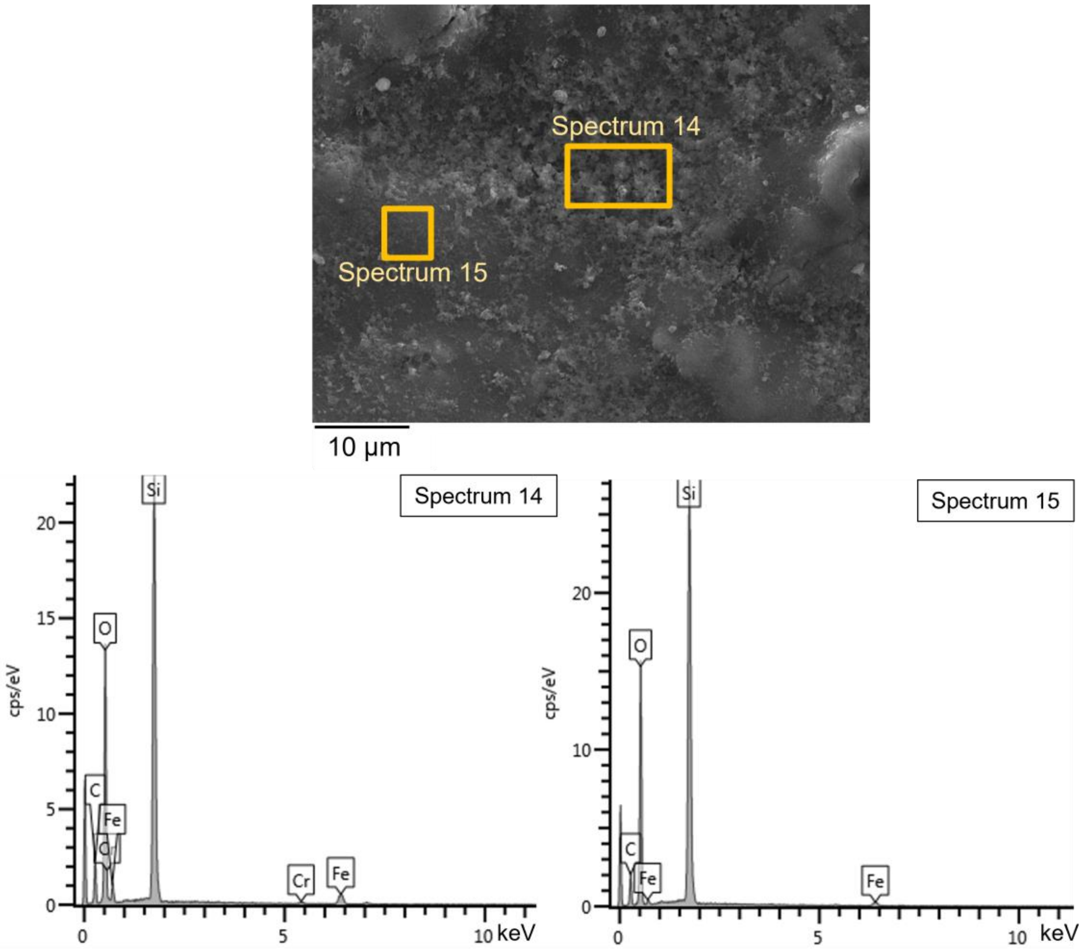

Figure S30. SEM image (2000x magnification) and EDS spectra of GSLP-Fe(0) D3 after use it in Cr(VI) removal.

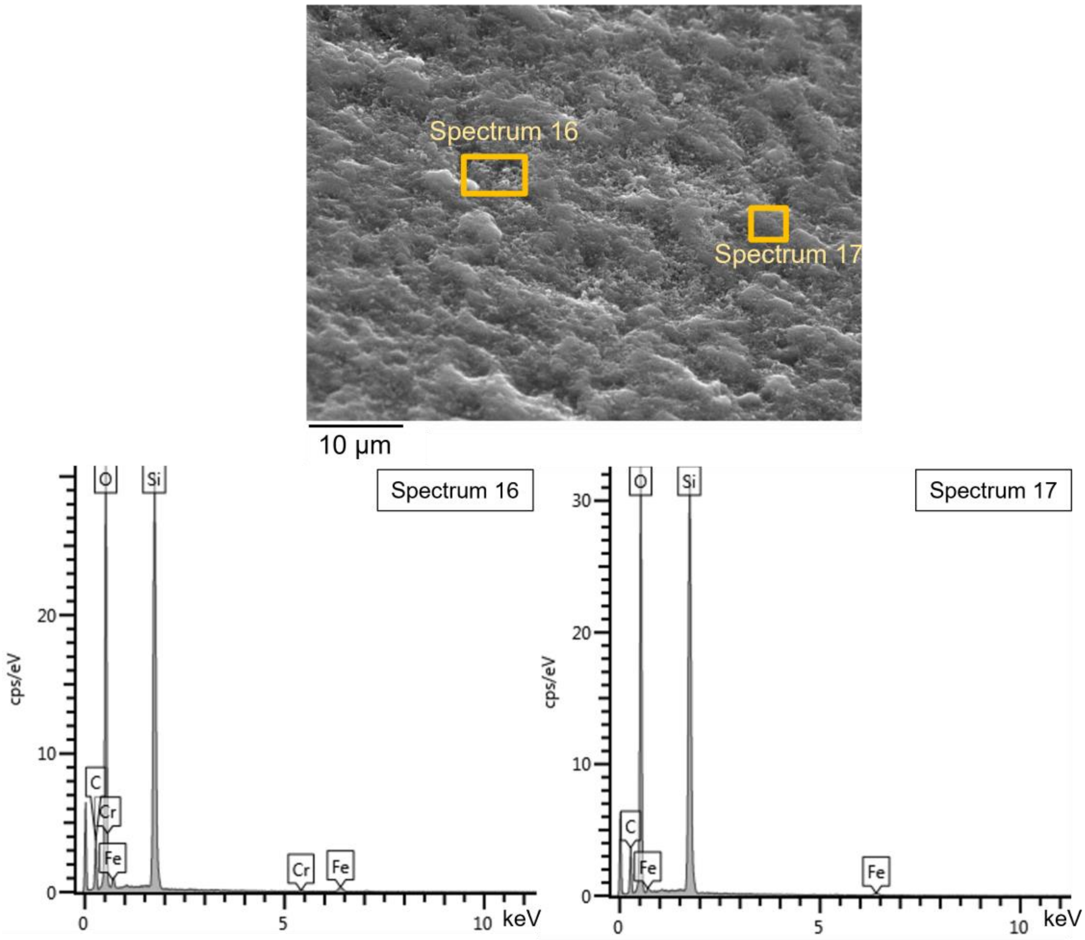

Figure S31. SEM image (2000x magnification) and EDS spectra of GSLP-Fe(0) D2 after use it in Cr(VI) removal.

Tables.

Table S1. Atomic and weight percentages of the main ions present in the EDS spectra of Fig. S4, S6, S8, S10, S12, S13, S26, S27, S30 and S31. The presence of C in samples not treated with eucalyptus extract, K and P is due to sample pre-treatments for analysis.

Element		Spectrum																
		1	2	3	4	5	6	7	8	9	10	11	12	13	14	15	16	17
% Atomic	C	42.7	23.8	21.9	30.8	33.2	28.7	29.5	38.9	35.7	30.9	38.8	-	-	-	-	-	-
	Si	9.4	20.3	19.8	11.7	18.8	19.3	13.1	19.0	6.7	19.1	9.9	-	-	-	-	-	-
	O	18.4	54.6	57.0	51.5	47.5	51.4	50.4	40.5	37.9	46.4	45.5	-	-	-	-	-	-
	Na	16.2	1.2	1.2	-	-	0.6	-	-	-	-	-	-	-	-	-	-	-
	Cl	13.3	0.1	0.1	-	-	-	-	-	-	-	-	-	-	-	-	-	-
	Fe	-	-	-	5.1	0.5	-	6.7	1.6	19.0	0.6	5.4	-	-	-	-	-	-
	Cr	-	-	-	-	-	-	-	-	-	-	-	-	-	-	-	-	-
	S	-	-	-	-	-	-	0.3	-	-	-	-	-	-	-	-	-	-
	P	-	-	-	0.6	-	-	-	-	0.7	-	0.4	-	-	-	-	-	-
	K	-	-	-	0.3	-	-	-	-	-	-	-	-	-	-	-	-	-
% Weight	C	26.8	16.3	14.9	20.2	23.3	19.8	18.5	26.9	18.6	21.5	26.1	22.6	16.9	22.7	20.4	19.8	19.3
	Si	13.7	32.3	31.6	17.8	30.8	31.1	19.3	30.7	8.1	30.9	15.6	33.1	29.2	28.5	32.1	24.1	54.6
	O	15.4	49.6	51.7	44.9	44.4	47.3	42.1	37.2	26.3	45.6	40.8	44.3	53.9	41.6	44.8	53.2	25.1
	Na	19.4	1.6	1.6	-	-	-	-	-	-	-	-	-	-	-	-	-	-
	Cl	24.7	0.2	0.2	-	-	-	-	-	-	-	-	-	-	-	-	-	-
	Fe	-	-	-	15.5	1.5	1.8	19.6	5.2	46.0	2.0	16.8	-	-	6.6	2.7	2.5	1.0
	Cr	-	-	-	-	-	-	-	-	-	-	-	-	-	0.6	-	0.4	-
	S	-	-	-	-	-	-	0.5	-	-	-	-	-	-	-	-	-	-
	P	-	-	-	1.0	-	-	-	-	1.0	-	0.7	-	-	-	-	-	-
	K	-	-	-	0.6	-	-	-	-	-	-	-	-	-	-	-	-	-

Table S2. Kinetic parameters obtained by the Boyd non-linear model (Eq. 2).

Metal	Kinetic parameters			Non-linear Boyd Model		
	Pellet diameter	c_i ($mg \cdot L^{-1}$)	pH	B	q	R^2
Fe(II)	D3	75	Nat	$47.5 \cdot 10^{-2} \pm 1.8 \cdot 10^{-2}$	15.35 ± 0.13	0.997
	D2	75	Nat	$57 \cdot 10^{-2} \pm 3 \cdot 10^{-2}$	17.40 ± 0.15	0.996
Cr(VI)	D3	50	1.2	$110 \cdot 10^{-3} \pm 6 \cdot 10^{-3}$	15.6 ± 0.2	0.986
	D3	50	0.8	$13.2 \cdot 10^{-2} \pm 1.0 \cdot 10^{-2}$	13.2 ± 0.2	0.985
	D3	100	0.8	$23 \cdot 10^{-2} \pm 3 \cdot 10^{-2}$	15.2 ± 0.3	0.917
	D3	75	0.8	$125 \cdot 10^{-3} \pm 9 \cdot 10^{-3}$	15.8 ± 0.2	0.983
	D2	50	1.2	$103 \cdot 10^{-3} \pm 8 \cdot 10^{-3}$	14.7 ± 0.2	0.982
	D2	50	0.8	$15.1 \cdot 10^{-2} \pm 1.2 \cdot 10^{-2}$	14.2 ± 0.3	0.986
	D2	75	0.8	$15.7 \cdot 10^{-2} \pm 1.6 \cdot 10^{-2}$	14.2 ± 0.3	0.967
